# Phosphorylation of PKCδ by FER tips the balance from EGFR degradation to recycling

**DOI:** 10.1083/jcb.201902073

**Published:** 2021-01-07

**Authors:** Ana Lonic, Freya Gehling, Leila Belle, Xiaochun Li, Nicole L. Schieber, Elizabeth V. Nguyen, Gregory J. Goodall, Robert G. Parton, Roger J. Daly, Yeesim Khew-Goodall

**Affiliations:** 1 Centre for Cancer Biology, An Alliance of SA Pathology and the University of South Australia, Adelaide, South Australia, Australia; 2 The Discipline of Medicine, Faculty of Health and Medical Sciences, University of Adelaide, Adelaide, South Australia, Australia; 3 Institute for Molecular Bioscience, The University of Queensland, St. Lucia, Queensland, Australia; 4 Cancer Program, Biomedicine Discovery Institute, and Department of Biochemistry and Molecular Biology, Monash University, Clayton, Victoria, Australia; 5 School of Biological Sciences, University of Adelaide, Adelaide, South Australia, Australia; 6 Centre for Microscopy and Microanalysis, The University of Queensland, St. Lucia, Queensland, Australia; 7 Department of Molecular and Cellular Biology, School of Biological Sciences, University of Adelaide, Adelaide, South Australia, Australia

## Abstract

Receptor degradation terminates signaling by activated receptor tyrosine kinases. Degradation of EGFR occurs in lysosomes and requires the switching of RAB5 for RAB7 on late endosomes to enable their fusion with the lysosome, but what controls this critical switching is poorly understood. We show that the tyrosine kinase FER alters PKCδ function by phosphorylating it on Y374, and that phospho-Y374-PKCδ prevents RAB5 release from nascent late endosomes, thereby inhibiting EGFR degradation and promoting the recycling of endosomal EGFR to the cell surface. The rapid association of phospho-Y374-PKCδ with EGFR-containing endosomes is diminished by PTPN14, which dephosphorylates phospho-Y374-PKCδ. In triple-negative breast cancer cells, the FER-dependent phosphorylation of PKCδ enhances EGFR signaling and promotes anchorage-independent cell growth. Importantly, increased Y374-PKCδ phosphorylation correlating with arrested late endosome maturation was identified in ∼25% of triple-negative breast cancer patients, suggesting that dysregulation of this pathway may contribute to their pathology.

## Teaser

Lonic et al. show that phosphorylation of Y374-PKCδ by FER arrests the maturation of early to late endosomes by inhibiting the release of RAB5 from nascent late endosomes. This promotes EGFR recycling and sustained signaling in triple-negative breast cancer.

## Introduction

Receptor tyrosine kinases (RTKs) are critical regulators of many cellular processes, including cell proliferation, differentiation, metabolism, migration, and invasion. Diseases as diverse as diabetes and cancer have causal links to mutations in RTKs or to their aberrant expression or localization (Lemmon and Schlessinger, 2010). Unfortunately, the use of single RTK–targeted drugs has largely failed to provide durable response for cancer patients due to either intrinsic or developed resistance arising from a range of mechanisms. For example, it is now becoming clear that adaptive resistance to mitogen-activated protein kinase kinase enzyme inhibitors (MEK-Is), anaplastic lymphoma kinase inhibitors (ALK-Is), or B-Raf proto-oncogene, serine/threonine kinase inhibitors (BRAF-Is) in many different cancers is driven by the up-regulation of multiple RTKs rendering inhibition of any one RTK ineffective in overcoming the resistance (Caunt et al., 2015; Duncan et al., 2012; Dardaei et al., 2018). Furthermore, in cancers with loss of a common negative feedback inhibitor of RTK activation such as loss of PTPN12 expression, single-agent RTK inhibition is ineffective because of hyperactivation of multiple RTKs (Sun et al., 2011). The important observation is that in both cases, the use of combinations of RTK inhibitors or broad-range RTK inhibitors to target multiple RTKs simultaneously can be an effective strategy. In triple-negative breast cancer (TNBC) with loss of PTPN12, treatments using a combination of RTK inhibitors are effective in mediating cell death, including in chemorefractory cancers (Nair et al., 2018). In many cancers, the unique changes to the kinome in response to each inhibitor, coupled with the variation in response by individual tumor cells to any one inhibitor (Duncan et al., 2012), makes it difficult to predict which RTKs will be up-regulated when the cancer becomes treatment resistant. It is therefore preferable to identify means that will universally inhibit signaling from the multiple RTKs expressed by the cancer cells. The effectiveness of this strategy was recently demonstrated by the resensitization of ALK-I– or MEK-I–resistant cancer cells to ALK-I (Dardaei et al., 2018) or MEK-I (Fedele et al., 2018) by inhibiting SHP2, a common downstream regulator of signals emanating from multiple RTKs, suggesting that identifying novel common control points for regulating signaling from multiple RTKs may provide new therapeutic targets to overcome resistance.

Two fundamental control points for RTK signaling are receptor activation and signal termination. Receptor activation occurs upon ligand binding to the RTK extracellular domain (ECD). The amount of receptor on the cell surface determines the maximum amplitude of signal that can be received and also influences the duration of signal reception when ligand is abundantly available. Signal termination occurs when endocytosed RTKs are transported to the lysosome and degraded (Wiley and Burke, 2001; Sorkin and von Zastrow, 2009; Miaczynska, 2013). However, a proportion of endocytosed RTKs can avoid degradation by being recycled back to the cell surface, where they can continue to be activated, thus extending the duration of signaling (Tomas et al., 2014). Consequently, the balance between the proportion of RTKs that is recycled back to the cell surface relative to that which is directed to the lysosome for degradation is an important determinant of the amplitude and duration of signaling.

Endosomal trafficking of RTKs is regulated by mechanisms that are both inherent to and independent of the properties of the receptors (Honegger et al., 1990; Barbieri et al., 2000; Alwan et al., 2003; Miaczynska, 2013; Tomas et al., 2015; Tan et al., 2016; Francavilla et al., 2016; Schmid, 2017). Following endocytosis triggered by ligand binding, RTKs are transported to the early endosomes, the main sorting station, where they are sorted either for recycling back to the plasma membrane or to the lysosome for degradation (Huotari and Helenius, 2011). RTKs that are destined for degradation are sorted into intraluminal vesicles (ILVs) within the early endosome during the progression from early to late endosomes, enabling the delivery of the RTKs to the lysosome. Therefore, the rate of early to late endosome maturation may constitute a critical receptor-independent mechanism to modulate the balance between recycling and degradation of endocytosed receptors.

The recruitment of specific ras-associated binding proteins (RABs) is essential for defining the function of specific endosomal compartments (Zerial and McBride, 2001; Gruenberg, 2001; Huotari and Helenius, 2011). Following endocytosis, RAB5 is recruited to the early endosomes, but the maturation of early to late endosomes requires a RAB5 to RAB7 switch on the endosome that is vital to the subsequent delivery of cargo to the lysosome (Rink et al., 2005; Poteryaev et al., 2010; Vonderheit and Helenius, 2005). Slowing the rate of RAB5 to RAB7 switching would provide greater opportunity for recycling from the early endosome to continue and thereby prolong and/or increase signaling from RTKs. While many of the components involved in the recruitment, activation, and inactivation of RAB5 and RAB7 have been identified (Poteryaev et al., 2010; Haas et al., 2005; Jimenez-Orgaz et al., 2018; Seaman et al., 2009), a crucial gap remains in our understanding of how recruitment of RAB7 and subsequent shedding of RAB5 is coordinated, and it is this switch that ultimately modulates the balance between degradation and recycling of RTKs.

We have previously identified the protein kinase PKCδ (PRKCD), phosphorylated on tyrosine 374 (Y374), as a substrate of the nonreceptor tyrosine phosphatase and tumor suppressor PTPN14 (Pez or PTPD2) and have shown that reduced expression or catalytic activity of PTPN14 leads to increased cell surface expression of the RTKs vascular endothelial growth factor receptor 3 (VEGFR3 or FLT4) in primary lymphatic endothelial cells and epidermal growth factor receptor (EGFR) in breast cancer cells (Belle et al., 2015). Here we identify the tyrosine kinase FER as the kinase that phosphorylates PKCδ on Y374 and show that phospho-Y374-PKCδ stabilizes a normally transient RAB5-RAB7–positive endosome population to shift the balance from RTK degradation to recycling, with potential consequences for the survival and growth of a subset of triple-negative and HER2^+^ breast cancers.

## Results

### Phosphorylation of PKCδ on Y374 promotes recycling and reduces degradation of EGFR

**Figure S1.**
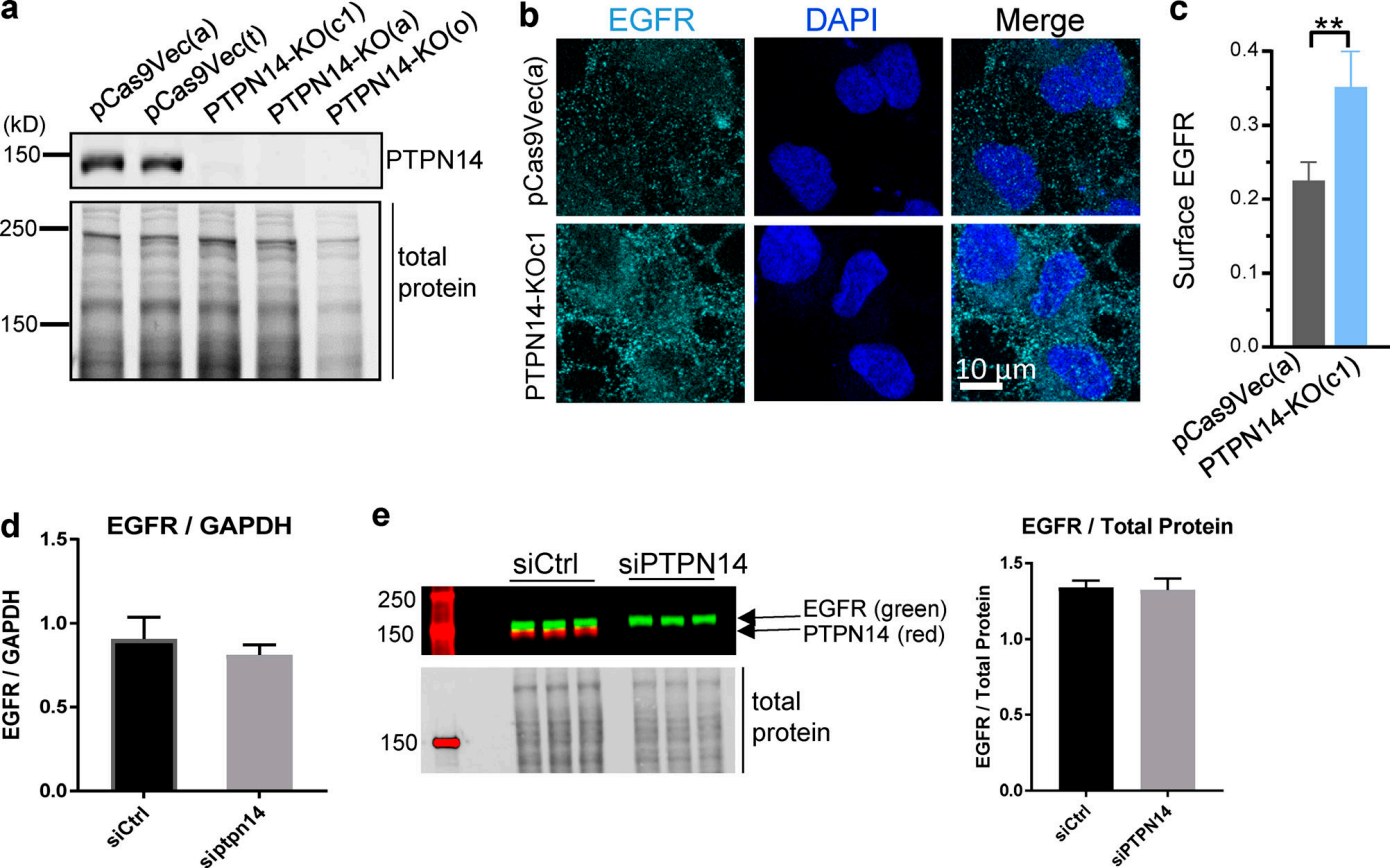
**Loss of PTPN14 enhances cell surface expression of EGFR but not EGFR transcript or total protein. (a)** Representative Western blot showing loss of PTPN14 in three independent PTPN14-KO BT-549 cell clones. **(b and c)** Cell surface EGFR measured by cell surface staining of EGFR sequentially labeled with mouse EGFR-ECD Ab followed by anti-mouse–Alexa Fluor 594 secondary Ab (false-colored green), showing representative confocal image (b) and quantitation of surface EGFR (c; mean fluorescence intensity) from confocal images (mean ± SD; *n* = 2 independent experiments; minimum 85 cells per treatment condition per experiment; **, P < 0.01, two-tailed Student’s *t* test). This is a subset of the data shown in Fig. 2 c. **(d)** Quantitative RT-PCR showing EGFR mRNA levels, relative to GAPDH (mean ± SD; *n* = 3). **(e)** Western blot showing EGFR and PTPN14 protein levels (left) and quantified relative to total protein (right; mean ± SD; *n* = 3).

The tyrosine phosphatase PTPN14 has been shown to limit levels of EGFR on the surface of breast cancer cells, potentially through dephosphorylation of the phospho-Y374 form of PKCδ (henceforth designated pY374-PKCδ; Belle et al., 2015), but how this affects intracellular trafficking of the receptor has not been determined. To address this, we first verified that loss of PTPN14 by CRISPR-Cas9–mediated knockout (KO) increases steady-state cell surface levels of EGFR in the absence of ligand (Fig. S1, a–c) and that depletion of PTPN14 did not result in a substantial overall change in total EGFR expression. Quantitative PCR for EGFR mRNA and Western blotting for total EGFR protein levels showed that the increased cell surface EGFR expression was not accounted for by increased EGFR synthesis (Fig. S1, d and e), indicating that the effect of PTPN14 in the absence of ligand is the result of altered trafficking.

**Figure 1.**
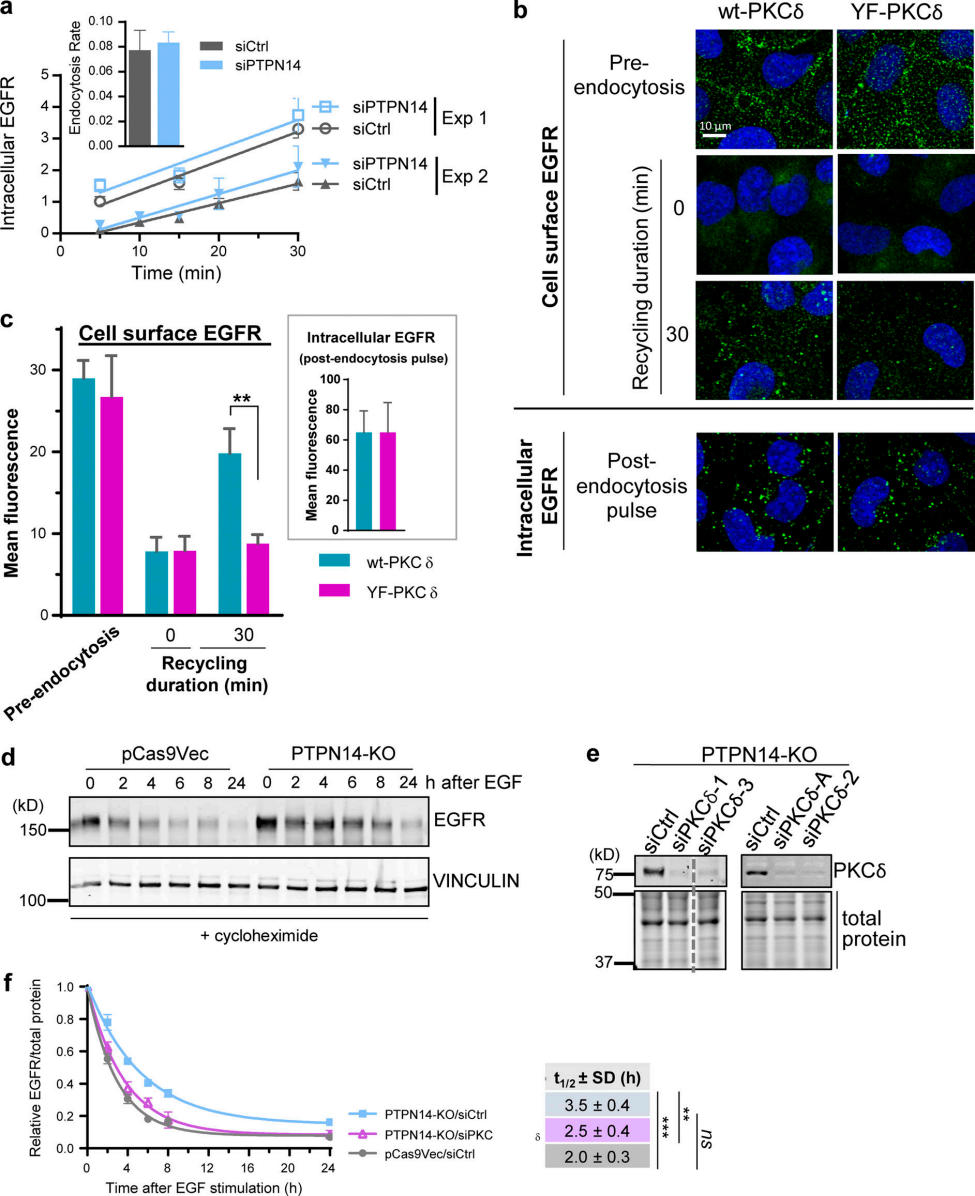
**pY374-PKCδ regulates EGFR recycling and degradation but not endocytosis. (a)** Time course of EGFR endocytosis, induced by addition of EGF (20 ng/ml), in BT-549 cells transiently transfected with either control (siCtrl) or PTPN14 (siPTPN14) siRNA (mean fluorescence intensity ± SD; *n* = 3 replicates) with linear regression line of the time course. Two independent experiments are shown. Inset: Rates of endocytosis (change in mean fluorescence intensity over time ± range; *n* = 2) calculated by linear regression analysis of each time course. **(b and c)** Phosphorylation on Y374 on PKCδ is required to promote EGFR recycling in PTPN14-KO cells. BT-549 cells overexpressing either WT (wt) or Y374F-PKCδ (Fig. S2 b) were prelabeled with the mouse EGFR-ECD antibody in the presence of 20 ng/ml EGF (4°C), and endocytosis was initiated by incubation at 37°C. After stripping any EGFR-ECD antibody and EGF remaining on the cell surface with an acid wash at 4°C after the pulse of endocytosis, EGFR recycling was initiated by incubation at 37°C and allowed to continue for 30 min. Representative immunofluorescence micrographs show either cell surface EGFR in nonpermeabilized cells or intracellular EGFR in permeabilized cells as indicated (b) and quantified data from *n* = 2 independent experiments (c; mean ± SD; **, P < 0.01 using multiple *t* tests; Prism). **(d–f)** Loss of PTPN14 represses, whereas concomitant PKCδ knockdown derepresses, EGFR degradation in BT-549 breast cancer cells. Representative Western blot showing EGFR levels (top panel) and vinculin as a loading control (lower panel) in pCas9Vec control or PTPN14-KO cells, pretreated with cycloheximide for 30 min before stimulation with 100 ng/ml EGF for the indicated times (d). Representative Western blot showing PKCδ knockdown in PTPN14-KO cells (e) and quantitation of EGFR levels, relative to total protein loaded, at each time point after EGF stimulation (mean ± SD from *n* = 5 independent experiments for pCas9Vec/siCtrl and PTPN14-KO/siCtrl and *n* = 4 independent experiments using four different PKCδ siRNAs for PTPN14-KO/siPKCδ; f). The *t*_1/2_ ± SD of EGFR was obtained using a nonlinear one-phase decay fit of the time course; ***, P < 0.001; **, P < 0.01, Student’s *t* test.

**Figure S2.**
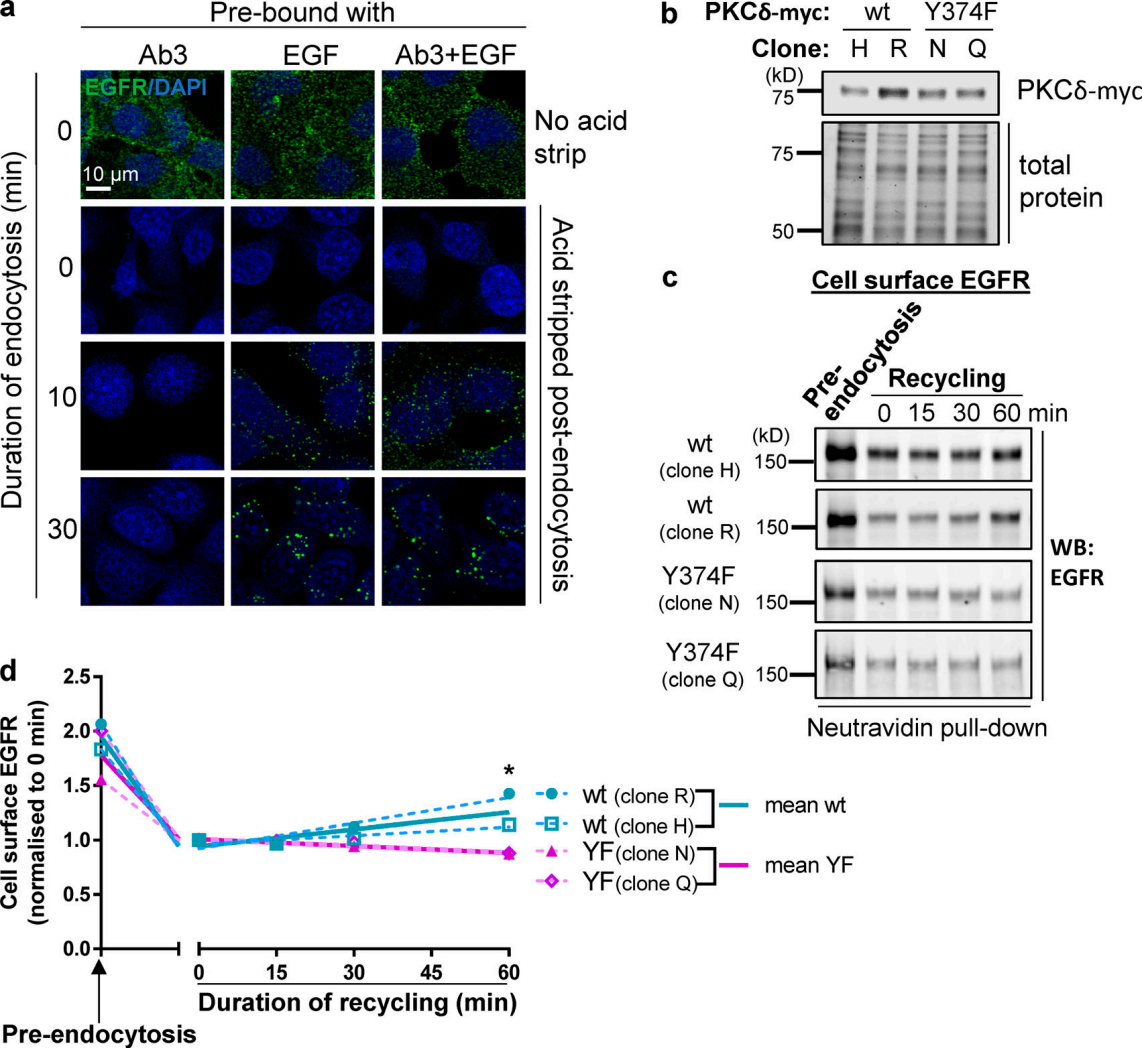
**Phosphorylation of Y374-PKCδ is required for EGFR recycling. (a)** The EGFR-ECD antibody (Ab3) alone does not induce endocytosis. Representative immunofluorescence micrographs showing cells prebound (at 4°C) with the EGFR-ECD antibody alone and EGF (20 ng/ml) alone or with the EGFR-ECD antibody in the presence of EGF. Cells were either fixed without inducing endocytosis (0 min, no acid strip) or induced to undergo endocytosis for various times, as indicated, by incubation at 37°C, followed by an acid wash to remove EGFR-ECD antibody and EGF remaining on the cell surface (acid strip) after endocytosis. Cells were then permeabilized and incubated with secondary Ab (cells prebound with Ab3, Ab3 + EGF) or with the Ab3 antibody followed by secondary antibody (cells prebound with EGF only) to detect endocytosed EGFR. **(b)** Representative Western blot showing two BT-549 clones overexpressing wt-PKCδ-myc and two clones overexpressing Y374F-PKCδ-myc. **(c and d)** EGFR recycling requires phosphorylation of Y374-PKCδ. BT-549 cells overexpressing wt (two clones, H and R) or Y374F-PKCδ (two clones, N and Q) were either surface biotinylated before inducing endocytosis (preendocytosis) or induced to undergo a pulse of endocytosis with EGF (20 ng/ml) and cell surface biotinylated after allowing recycling to commence for the duration indicated. The total cell surface biotinylated proteins were captured using NeutrAvidin beads and amount of biotinylated EGFR detected by Western blotting (WB) with an EGFR antibody (c) and quantified (d; graphs of individual clones [dashed lines] and the means of *n* = 2 wt and *n* = 2 Y374F-PKCδ clones [solid lines] plotted using segmental nonlinear regression analysis; *, P < 0.05, multiple *t* tests; Prism).

Because there are clear differences in the trafficking routes of EGF-bound EGFR compared with unliganded receptor (Sorkin and Goh, 2009; Baumdick et al., 2015), we next asked whether PTPN14 also regulates EGF-induced trafficking of EGFR and whether this is dependent on its substrate, pY374-PKCδ (PKCδ that is phosphorylated on Y374; Belle et al., 2015). Although PKCδ has been implicated in regulating the recycling of RTKs following ligand- or drug-induced endocytosis (Lladó et al., 2004, 2008; Park et al., 2013; Bailey et al., 2014), what regulates PKCδ function in RTK trafficking is not clear, and the role of phosphorylation of Y374 in EGFR trafficking or PKCδ function has not been investigated. We found that the rate of EGF-induced EGFR endocytosis is not significantly affected by depletion of PTPN14 (Fig. 1 a), suggesting that endocytosis is not affected by the level of pY374-PKCδ. To examine whether recycling of EGF-bound EGFR is regulated by pY374-PKCδ, we generated BT-549 cells with doxycycline (dox)-inducible myc-tagged PKCδ (WT-PKCδ-myc) or dox-inducible PKCδ rendered nonphosphorylatable at residue 374 by mutation to phenylalanine at this site (designated Y374F-PKCδ-myc). EGFR recycling following EGF-induced endocytosis was determined using two independent assays that directly measure the amount of EGFR on the cell surface. In the first approach, we used immunofluorescence microscopy to quantify cell surface EGFR following an EGF-stimulated endocytosis pulse and a 30-min recycling period, and found significantly more recycling in cells with WT-PKCδ than cells with Y374F-PKCδ (Fig. 1, b and c). Because this assay uses prebound EGFR-ECD antibody and EGF (at 4°C to prevent internalization) to track the fate of EGFR after binding EGF, controls were also performed to verify that the EGFR endocytosis observed was EGF dependent and not induced by the EGFR-ECD antibody alone. Indeed, endocytosis occurred only when EGF was present, and the kinetics and extent of endocytosis triggered by EGF were similar in the absence or presence of the prebound EGFR-ECD antibody (Fig. S2 a). These data suggest that recycling of EGFR after EGF-induced endocytosis is dependent on the phosphorylation of Y374-PKCδ.

To confirm the involvement of pY374-PKCδ in EGFR recycling, we used a second, independent approach to measure EGFR recycling, in which WT-PKCδ– and Y374-PKCδ–expressing cells were stimulated with EGF, and cell surface proteins were biotinylated after various periods of recycling. Biotinylated EGFR was then captured using NeutrAvidin beads and detected by Western blotting with an anti-EGFR antibody. Data obtained using the postrecycling biotinylation approach (Fig. S2, b–d) also showed significantly more recycling of EGFR with expression of WT-PKCδ, compared with Y374F-PKCδ, consistent with data obtained by the immunofluorescence assay above. Together, our data strongly indicate that phosphorylation of Y374-PKCδ is essential for EGFR recycling downstream of EGF binding.

**Figure S3.**
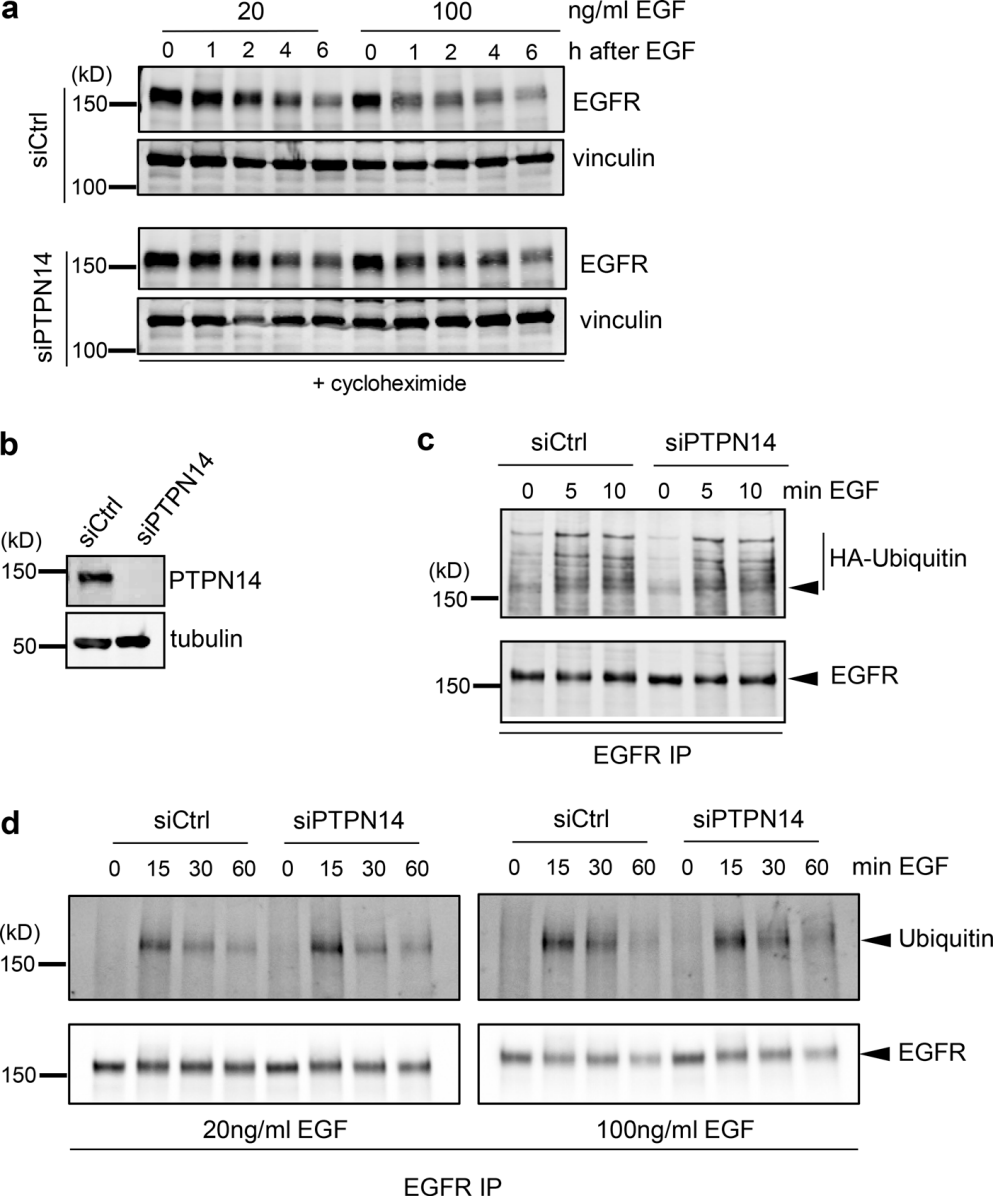
**Loss of PTPN14 reduces degradation of EGFR but does not affect EGFR ubiquitination. (a)** Representative Western blot showing EGFR levels (top panel) and total protein (represented by vinculin levels; lower panel) in siCtrl and siPTPN14 cells, pretreated with cycloheximide for 30 min before stimulation with 20 or 100 ng/ml EGF for the indicated times. **(b–d)** Ubiquitination of EGFR in control (siCtrl) or PTPN14 knockdown (siPTPN14) cells. Representative Western blots of PTPN14 expression from *n* = 3 independent experiments (b) and Western blots showing EGFR ubiquitination in cells transiently transfected with HA-ubiquitin and stimulated with 20 ng/ml (c and d) or 100 ng/ml (d) EGF for the indicated times, detected by EGFR immunoprecipitation (IP) immunoblotted with anti-HA or anti-ubiquitin Ab (upper panel) or with anti-EGFR Ab (lower panel). Arrowheads indicate position of EGFR.

Because endosomal trafficking events, including those modulated by PKCδ, can reciprocally modulate recycling and degradation (Lladó et al., 2004, 2008; Hoepfner et al., 2005), we also investigated whether PTPN14 and PKCδ affect degradation of EGFR in EGF-stimulated cells. The rate of decay of EGFR following EGF stimulation was significantly decreased with reduction/loss of PTPN14, an effect that was more pronounced with the addition of higher concentrations of EGF ligand (Fig. S3 a) and was observed both for siRNA-mediated PTPN14 knock-down (Fig. S3 a) and in PTPN14 KO cells (Fig. 1 d). Importantly, this decrease in EGFR degradation was restored to control levels when PKCδ was also knocked down (Fig. 1, e and f), indicating that the effect of PTPN14 loss on EGFR stability is mediated through its effect on PKCδ. Because casitas B-lineage lymphoma (CBL) protein–mediated ubiquitination of EGFR is essential for trafficking EGFR to lysosomes for degradation (Duan et al., 2003), we investigated whether the observed impedance of EGFR degradation might be due to a reduction in EGFR ubiquitination. However, loss of PTPN14 did not lead to reduced ubiquitination of EGFR (Fig. S3, b–d), suggesting that stabilization of EGFR was not brought about by inhibition of ubiquitination by PTPN14 loss/elevated pY374-PKCδ.

### The tyrosine kinase FER phosphorylates Y374-PKCδ to regulate endosomal trafficking

**Figure S4.**
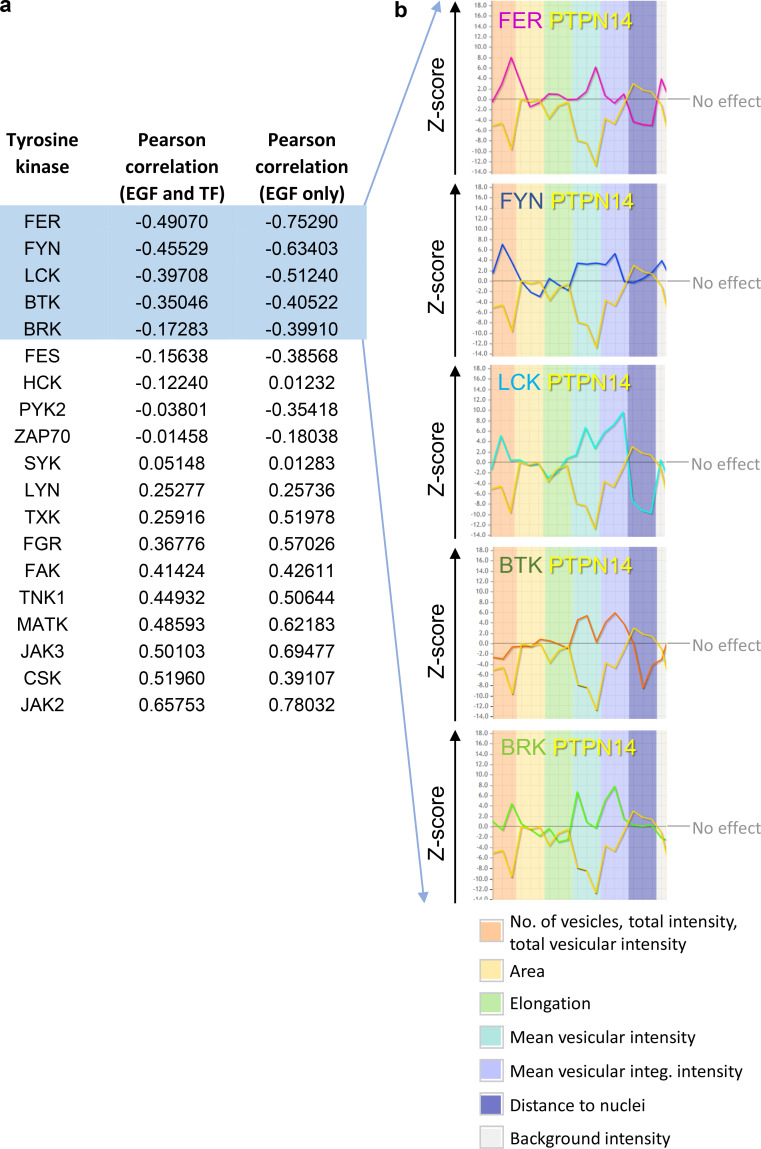
**In silico identification of TKs that oppose the action of PTPN14 in regulating endosomal phenotype. (a)** TKs identified from the endosomics database (Collinet et al., 2010; http://endosomics.mpi-cbg.de/) that play a role in determining endosomal phenotypes. Gene profile data were downloaded as a CSV file, and the candidate genes were ranked by negative Pearson correlation with PTPN14, across the EGF and Transferrin (TF) datasets, or against the EGF dataset only. **(b)** Z-score profiles of PTPN14 and the five most negatively correlated TKs for each of the EGF-specific endosomal parameters shown, generated using the endosomics database (Collinet et al., 2010).

**Figure 2.**
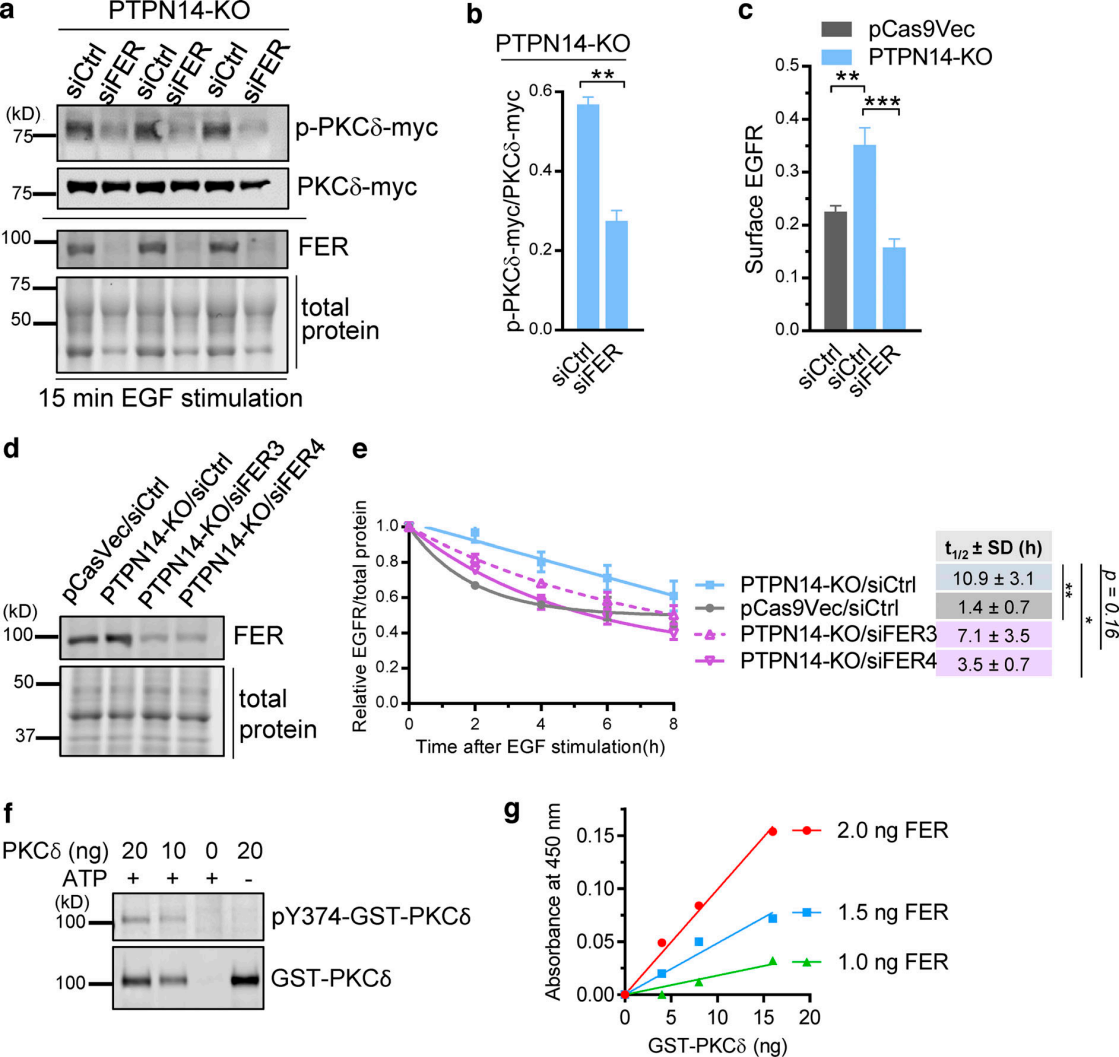
**FER phosphorylates Y374-PKCδ to promote cell surface EGFR and EGFR stabilization. (a and b)** FER deficiency reduces pY374-PKCδ. Western blot showing level of pY374-PKCδ-myc (p-PKCδ-myc) relative to PKCδ-myc expression in three different PTPN14-KO clones (c1, a, and o) overexpressing PKCδ-myc and transiently transfected with control (siCtrl) or a pool of four FER (siFER, Dharmacon Smartpool) siRNAs followed by stimulation with 20 ng/ml EGF for 15 min (a) and quantified data from the Western blot (b; mean ± SEM; *n* = 3 biological replicates; **, P < 0.01, Student’s *t* test), with pY374-PKCδ levels detected using the phospho-specific Ab to pY374-PKCδ. **(c)** FER deficiency represses EGFR cell surface expression to control levels, measured as mean fluorescence intensity in PTPN14-KO cells. Cell surface EGFR in pCas9Vec or PTPN14-KO cells transiently transfected with control (siCtrl) or a pool of four FER (siFER) siRNAs (means ± SEM quantified from *n* > 30 cells from one representative experiment of three; **, P < 0.01; ***, P < 0.001, Student’s *t* test). **(d and e)** FER deficiency restores EGFR degradation in PTPN14-KO cells. Representative Western blot of FER expression in pCas9Vec or PTPN14-KO cells that were transiently transfected with either siCtrl or two different FER siRNAs (siFER3 and siFER4) separately (d) and time course of EGFR expression after EGF stimulation (100 ng/ml), obtained by Western blotting, relative to total protein in pCas9Vec or PTPN14-KO cells that were transiently transfected with siCtrl, siFER3, or siFER4 (e). Cells were pretreated 30 min with cycloheximide before EGF stimulation. The *t*_1/2_ ± SD of EGFR was obtained using a nonlinear one-phase decay fit of the time course; **, P < 0.01; *, P < 0.05; Student’s *t* test from *n* = 4 independent experiments. **(f and g)** In vitro phosphorylation of recombinant GST-PKCδ on Y374 by recombinant FER. Western blot of the indicated amounts of GST-PKCδ after incubation with recombinant FER in the presence or absence of ATP, as indicated, and blotted with either the pY374-PKCδ phospho-specific Ab or total PKCδ Ab (f) and a colorimetric assay of pY374-PKCδ using constant amounts of recombinant PKCδ with varying amounts of recombinant FER and vice versa (g). Data representative of three independent experiments.

Our data indicate that the phosphorylation status of Y374-PKCδ is a critical regulatory step for endosomal trafficking of EGFR. However, the upstream kinase that phosphorylates PKCδ at this site is unknown. To address this knowledge gap, we mined an endosomics database (Collinet et al., 2010) for all tyrosine kinases whose knockdown affected endosome phenotypes. 19 TKs were identified (Fig. S4 a). We then refined our search by making the assumption that any TK that opposes the action of PTPN14 (the tyrosine phosphatase that dephosphorylates pY374-PKCδ) in this context would exhibit an endosomal phenotype profile that is the inverse of that for PTPN14. We ranked the TKs using Pearson correlation analysis (Fig. S4 a), comparing the endosome profile of each TK knockdown with that of the PTPN14 knockdown profile (Fig. S4 b), with the top-ranked candidate having the most negative correlation. We then knocked down the top candidate TK, FER, in PTPN14-KO cells, to determine whether loss of FER could reverse the phosphorylation of Y374-PKCδ, the increased cell surface EGFR expression, and the reduced degradation of EGFR induced by loss of PTPN14. Knockdown of FER using a pool of four siRNAs significantly reduced pY374-PKCδ levels (Fig. 2, a and b) and returned EGFR cell surface expression levels to that of the pre–PTPN14-KO cells (Fig. 2 c), verifying that FER is the TK that phosphorylates Y374-PKCδ and opposes the action of PTPN14 in this context. Using two different siRNAs deconvolved from the pool of four to knock down FER in PTPN14-KO cells, which have elevated pY374-PKC (Fig. 2 d), the degradation rate of EGFR was also significantly increased (Fig. 2 e), as was seen when PKCδ itself was knocked down (Fig. 1, e and f). To confirm that FER directly phosphorylates Y374-PKCδ, we used recombinant FER and PKCδ and demonstrated in vitro phosphorylation of Y374-PKCδ by FER that was detected by the pY374-PKCδ Ab (Fig. 2, f and g).

### Phosphorylation of Y374-PKCδ by FER promotes mitogenic signaling and anchorage-independent growth in breast cancer cells

**Figure 3.**
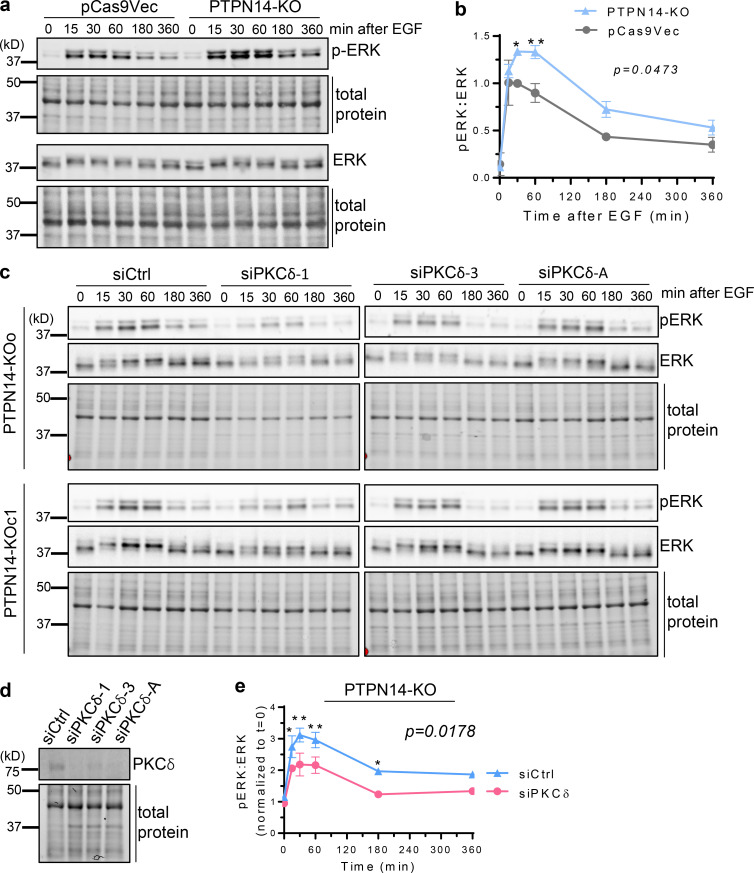
**pY374-PKCδ enhances ERK activation downstream of EGFR. (a and b)** ERK activation after stimulation with EGF (20 ng/ml), for the indicated times, was analyzed by Western blotting with rabbit anti-phospho-ERK and mouse anti-ERK Abs in pCas9Vec and PTPN14-KO cells (a) and quantified (b; mean ± SD from *n* = 2 pCas9Vec and *n* = 2 PTPN14-KO clones, P = 0.0473 using two-way ANOVA followed by Sidak’s multiple comparisons test for each time point; *, P < 0.05; **, P < 0.01). **(c)** PTPN14-KO cells transiently transfected with control (siCtrl) or three different PKCδ (siPKCδ) siRNAs with phospho-ERK and total ERK Western blots shown. (**d and e)** PKCδ blots (d) and ratio of pERK to total ERK blots quantified (e; *n* = 2 PTPN14-KO clones, each transfected with siCtrl and three different PKCδ siRNAs (averaged for each clone); P = 0.0178 using two-way ANOVA followed by Sidak’s multiple comparisons test for each time point; *, P < 0.05; **, P < 0.01).

**Figure 4.**
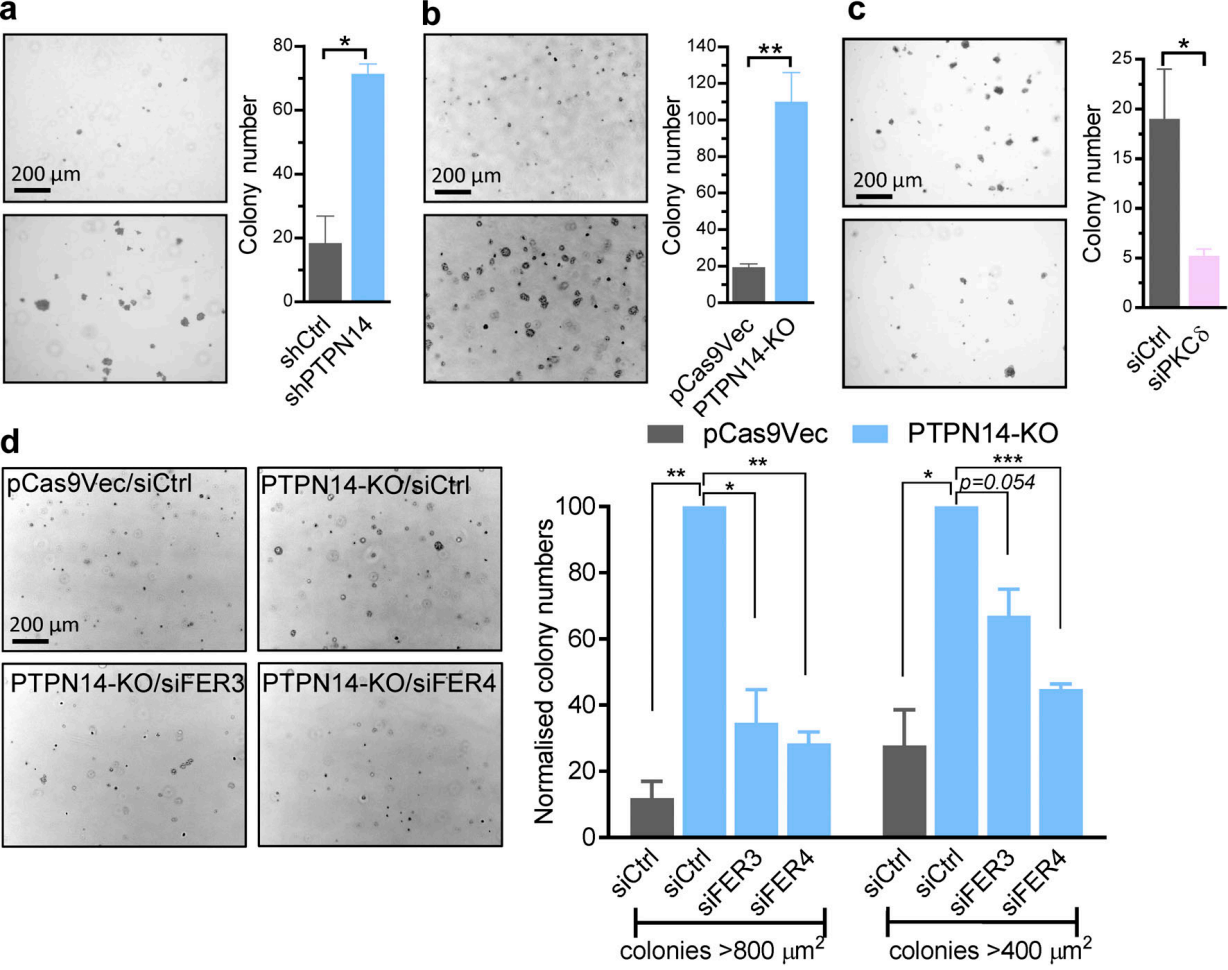
**PKCδ and FER enhance cancer cell growth downstream of EGFR activation. (a–d)** Colony-forming potential of breast cancer cell lines in the presence of EGF (20 ng/ml), showing representative micrographs of colonies (left) and quantified data (right) of control (shCtrl) or PTPN14 knockdown (shPTPN14) MDA-MB-231(LM2) cells (day 16 colonies, *n* = 2; a), pCas9Vec or PTPN14-KO BT-549 cells (day 7 colonies, *n* = 3; b), BT-549 cells transfected with control (siCtrl) or PKCδ (siPKCδ) siRNA (day 8 colonies, *n* = 3; c), and pCas9Vec or PTPN14-KO BT-549 cells transiently transfected with siCtrl, siFER3, or siFER4 (day 7 colonies, *n* = 3; d; mean ± SEM; *, P < 0.05; **, P < 0.01; ***, P < 0.001 using Student’s *t* test).

Our data, which link increased pY374-PKCδ levels (regulated by the opposing actions of FER and PTPN14) with increased EGFR recycling to the cell surface at the expense of receptor degradation, led us to investigate the effect of increased pY374-PKCδ on signaling downstream of EGFR. Cells with increased pY374-PKCδ due to the loss of PTPN14 exhibited a significant increase in the magnitude of ERK activation following ligand stimulation (Fig. 3, a and b), and this effect was mitigated with concomitant knockdown of PKCδ (Fig. 3, c–e). These effects on signaling were reflected functionally by an increase in colony-forming potential in soft agar (Fig. 4 a; PTPN14 knockdown in MDA-MB-231(LM2); Fig. 4 b; PTPN14-KO), both in the numbers of colonies and in the size of individual colonies formed. In contrast, cells lacking PKCδ showed impaired colony-forming potential (Fig. 4 c), suggesting that PKCδ is critical for anchorage-independent growth. To verify that phosphorylation of Y374-PKCδ by FER is also important for anchorage-independent growth, we knocked down FER in PTPN14-KO cells. Reducing FER expression overcame the increase in colony number brought about by increased pY374-PKCδ levels in PTPN14 KO cells (Fig. 4 d). The reduction in numbers of large colonies (>800 µm^2^) when FER is reduced also suggests that pY374-PKCδ promotes proliferation. Taken together, these data indicate that alterations in the level of pY374-PKCδ, regulated by the opposing actions of the tyrosine kinase FER and the tyrosine phosphatase PTPN14, modulate EGFR signaling and the growth of breast cancer cells.

### pY374-PKCδ and FER inhibit the RAB5-RAB7 switch essential for late endosome maturation

**Figure 5.**
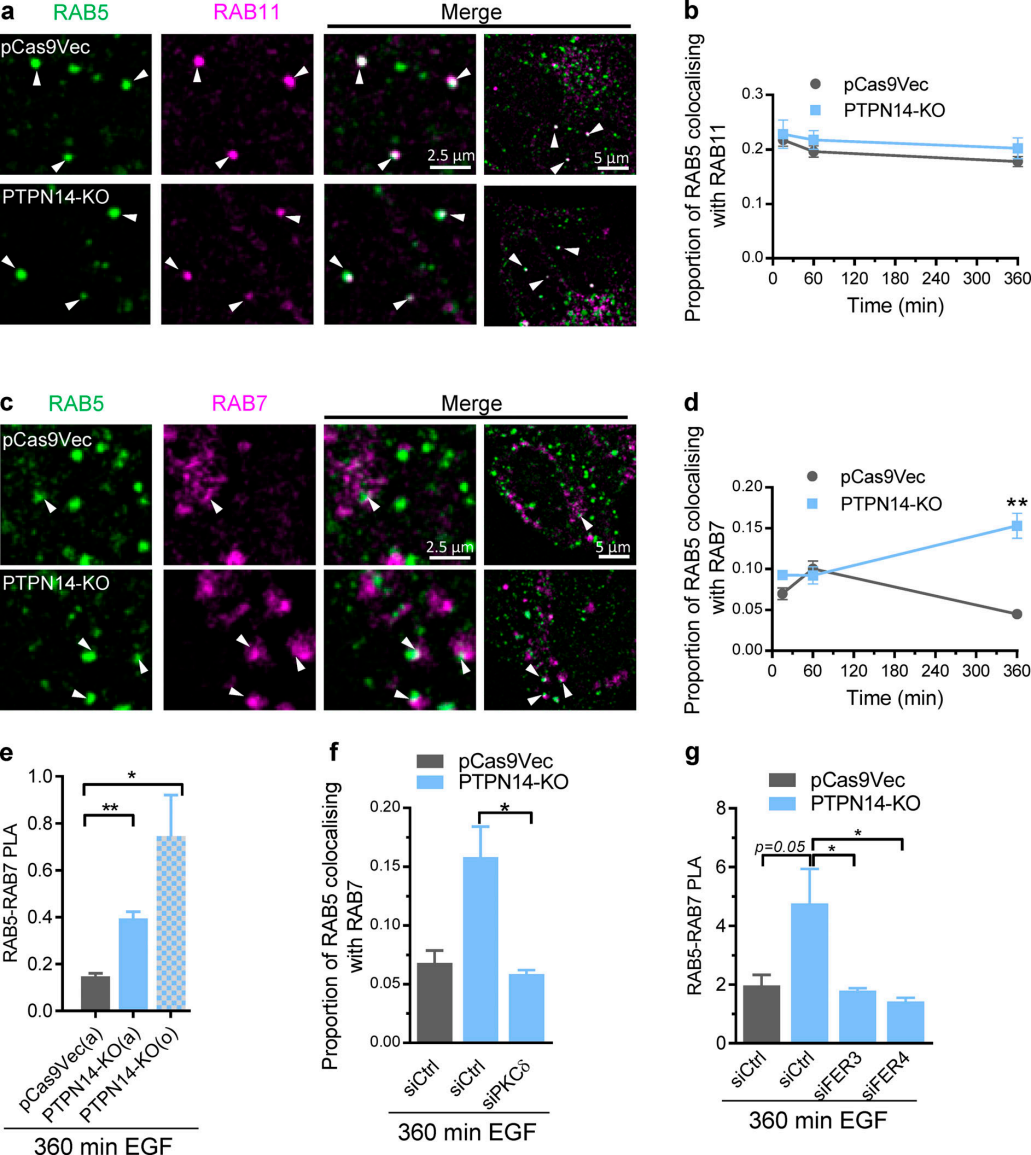
**pY374-PKCδ and FER inhibit release of RAB5 from the nascent late endosome. (a–d)** Time course of RAB11 and RAB7 recruitment to RAB5-positive endosomes. Representative confocal images of the indicated RAB immunofluorescence 360 min after initiating trafficking by EGF (20 ng/ml) stimulation in pCas9Vec or PTPN14-KO cells (a and c; arrowheads indicate colocalization) and quantification from confocal images of the colocalization of RAB5 (Alexa Fluor 488) immunofluorescence with either RAB11 (b; Alexa Fluor 594) or RAB 7 (d; Alexa Fluor 594) immunofluorescence at the indicated times after EGF stimulation (mean ± SEM; *n* > 30 cells; **, P < 0.01 using Student’s *t* test). **(e)** PLAs of RAB5 and RAB7 after 360-min EGF (20 ng/ml) stimulation in pCas9Vec or PTPN14-KO (two different clones, a and o) cells (mean ± SEM; *n* = 3; *, P < 0.05; **, P < 0.01 using Student’s *t* test). **(f)** Colocalization of RAB5 and RAB7 immunofluorescence 360 min after EGF (20 ng/ml) stimulation to induce trafficking in pCas9Vec or PTPN14-KO cells transiently transfected with control (siCtrl) or PKCδ (siPKCδ) siRNAs, quantified from confocal images (not shown; mean ± SEM; *n* > 30 cells; *, P < 0.05, using Student’s *t* test). **(g)** PLA of RAB5 and RAB7 after 360-min EGF (20 ng/ml) stimulation in pCas9Vec or PTPN14-KO transiently transfected with siCtrl, siFER3, or siFER4 siRNAs after 360 min of EGF stimulation to induce trafficking (mean ± SEM; *n* = 3; *, P < 0.05 using Student’s *t* test).

In the absence of evidence to suggest that the stabilization of EGFR caused by increased pY374-PKCδ levels is due to direct effects on EGFR itself, and because loss of PTPN14 has effects on the cell surface expression of multiple RTKs (Belle et al., 2015), we sought to establish whether pY374-PKCδ controls EGFR stability by controlling the trafficking machinery. During RTK trafficking, there is dynamic recruitment and shedding of different RABs from the endosomes as they mature. For example, following endocytosis of RTKs, RAB5 is rapidly recruited to the endocytic vesicles and is involved in their fusion to the early endosomes, which is followed by further recruitment of RAB5 to the early endosomes (Zerial and McBride, 2001; Sönnichsen et al., 2000). Recruitment of RAB11 to the RAB5-positive early endosomes initiates recycling, whereas recruitment of RAB7 initiates maturation of early endosomes to late endosomes and the path to degradation. We sought to ascertain whether pY374-PKCδ levels affected the association of RAB7 or RAB11 with RAB5-positive early endosomes by characterizing the kinetics of colocalization of RAB5 with either RAB7 or RAB11 after EGF stimulation. PTPN14 deficiency and the consequent change in levels of pY374-PKCδ did not significantly affect the kinetics of association between RAB5 and RAB11 (Fig. 5, a and b). In contrast, increasing pY374-PKCδ via PTPN14 loss prolonged colocalization of RAB5 with RAB7 (Fig. 5, c and d). We also verified the increased colocalization of RAB5 with RAB7 by proximity ligation assays (PLAs), an assay that generates a signal only when two proteins of interest are located no more than 30–40 nm apart, in two different PTPN14-KO clones (Fig. 5 e), suggesting that RAB5 and RAB7 are likely to be present on the same endosome. This is of particular interest, as the association of RAB7 with RAB5-positive early endosomes is normally transient due to the obligatory release of RAB5 after RAB7 recruitment (Rink et al., 2005; Huotari and Helenius, 2011). The data therefore suggest that elevating pY374-PKCδ levels may prevent the release of RAB5 from nascent late endosomes formed after recruitment of RAB7. The importance of Y374-PKCδ phosphorylation in the sustained association of RAB5 with RAB7 in PTPN14-KO cells is illustrated by the reduction of RAB5-RAB7 colocalization back to pre-PTPN14-KO levels when either PKCδ or FER expression was reduced using siRNAs targeting these proteins (Fig. 5, f and g).

### pY374-PKCδ and PTPN14 are sequentially recruited to EGFR-containing endosomes

**Figure 6.**
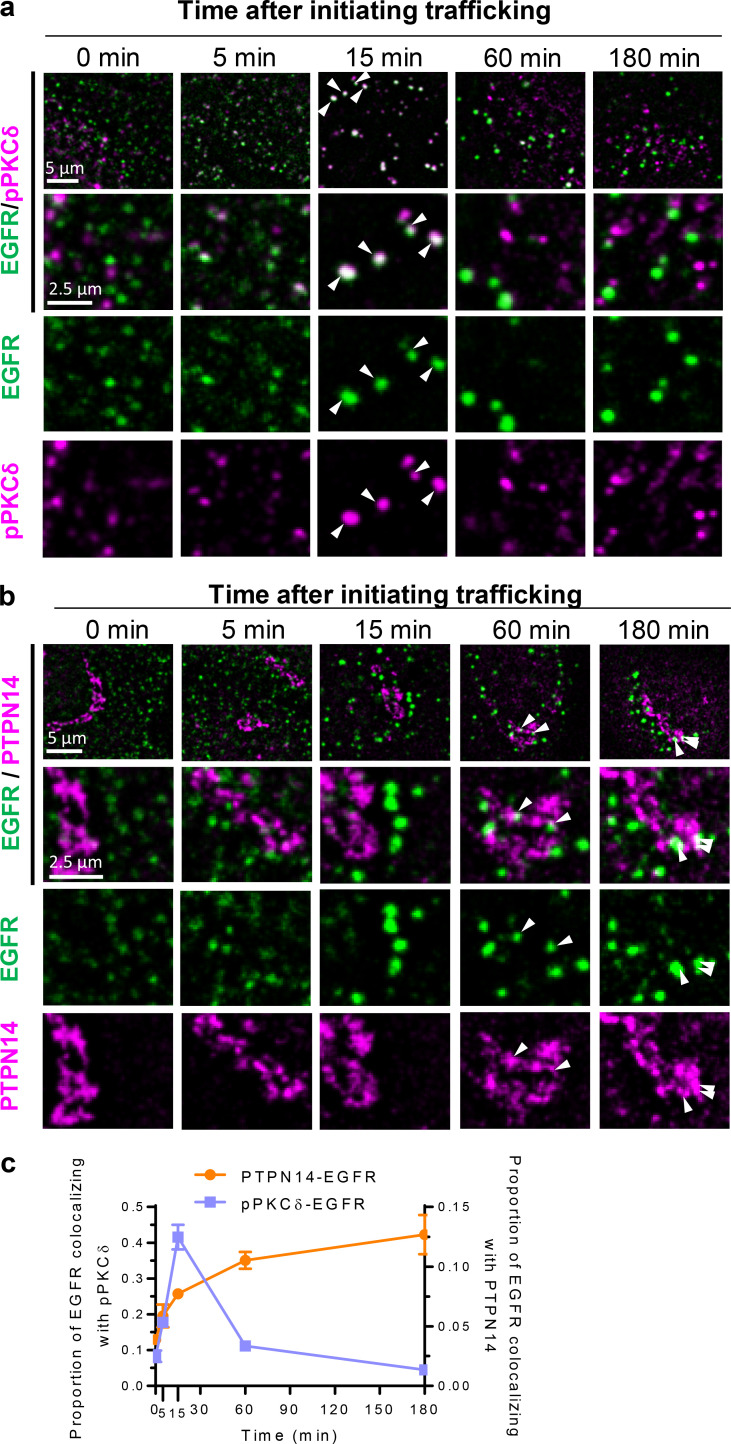
**pY374-PKCδ and PTPN14 are sequentially recruited to EGFR-containing endosomes after EGF stimulation.** Cell surface EGFR (SUM149PT cells) was labeled with EGFR-ECD antibody before inducing endocytosis with EGF (20 ng/ml). After fixation, EGFR was labeled with Alexa Fluor 594 while all other proteins shown were labeled with Alexa Fluor 488 at the indicated times after fixation. **(a–c)** Time course of colocalization of EGFR-containing endosomes (EGFR, Alexa Fluor 594, false-colored green) with pY374-PKCδ or PTPN14 (Alexa Fluor 488, false-colored red) as indicated. Representative low- and high-magnification and split-color confocal micrographs of EGFR-containing endosomes costained with either pY374-PKCδ (a) or PTPN14 (b) as indicated at the indicated times after initiating EGFR trafficking (arrowheads indicate colocalization), and time course of colocalization of EGFR-containing endosomes with either pY374-PKCδ or PTPN14 quantified from confocal micrographs (c; mean ± SEM; *n* > 30 cells per time point).

**Figure 7.**
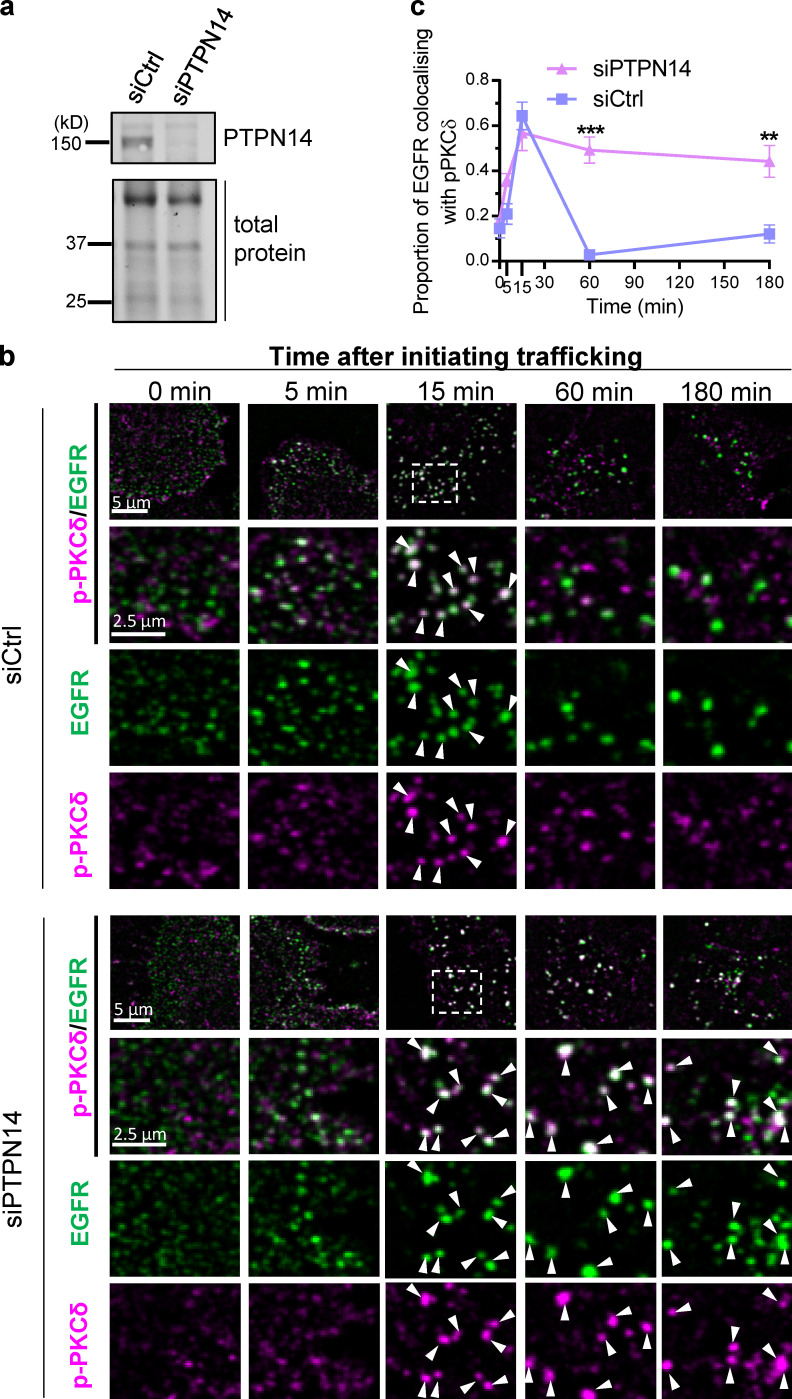
**PTPN14 deficiency prolongs association of pY374-PKCδ with EGFR-containing endosomes. (a)** Western blot showing PTPN14 expression in SUM149PT cells 48 h after transfection with control (siCtrl) or PTPN14 siRNA (siPTPN14). **(b and c)** Time course of colocalization of EGFR-containing endosomes (EGFR, Alexa Fluor 594, false-colored green) with pY374-PKCδ (Alexa Fluor 488, false-colored red) in siCtrl- or siPTPN14-transfected SUM149PT cells. Representative low- and high-magnification and split-color confocal micrographs of EGFR-containing endosomes costained with pY374-PKCδ at the indicated times after initiating EGFR trafficking with EGF (20 ng/ml; arrowheads indicate colocalization; b), and time course of colocalization of EGFR-containing endosomes with pY374-PKCδ in siCtrl- or siPTPN14-transfected cells, quantified from confocal micrographs (c; mean ± SEM; *n* > 30 cells per time point; **, P < 0.01; ***, P < 0.001 using two-way ANOVA followed by Sidak’s multiple comparisons test for each time point).

So far, our data suggested that pY374-PKCδ inhibits RAB5 release from the nascent late endosome and compromises the degradation of cargo carried by the endosomes. We hypothesized that following EGF stimulation, pY374-PKCδ may be associated with early endosomes carrying the EGFR and that the subsequent dephosphorylation of pY374-PKCδ by PTPN14 may be necessary to enable the completion of maturation from early to late endosomes, and subsequent lysosomal fusion. To explore this, we examined the kinetics of association of pY374-PKCδ and PTPN14 with EGFR-containing endosomes. pY374-PKCδ rapidly associated with internalized EGFR-containing endosomes after EGF stimulation, with peak association evident at 15 min after initiation of trafficking. This was followed by a relatively rapid loss of pY374-PKCδ from EGFR-containing endosomes (Fig. 6, a and c). The association of PTPN14 with EGFR-containing endosomes, in contrast, occurred later, with peak association at ≥60 min after initiation of trafficking (Fig. 6, b and c), coinciding with decreased association of pY374-PKCδ with the EGFR-containing endosomes. The kinetics of association of pY374-PKCδ and PTPN14 with endocytosed EGFR (Fig. 6 c) are consistent with our model of rapid recruitment of pY374-PKCδ to the early endosomes, which is permissive for recycling, followed by the later arrival of PTPN14 to dephosphorylate the endosome-associated pY374-PKCδ, allowing release of RAB5 from the nascent late endosome and a switch from recycling to degradation of EGFR. As predicted by this model, knockdown of PTPN14 led to prolonged association of pY374-PKCδ with endocytosed EGFR (Fig. 7, a–c), confirming that recruitment of PTPN14 is indeed required for down-regulating pY374-PKCδ associated with EGFR-containing endosomes.

**Figure 8.**
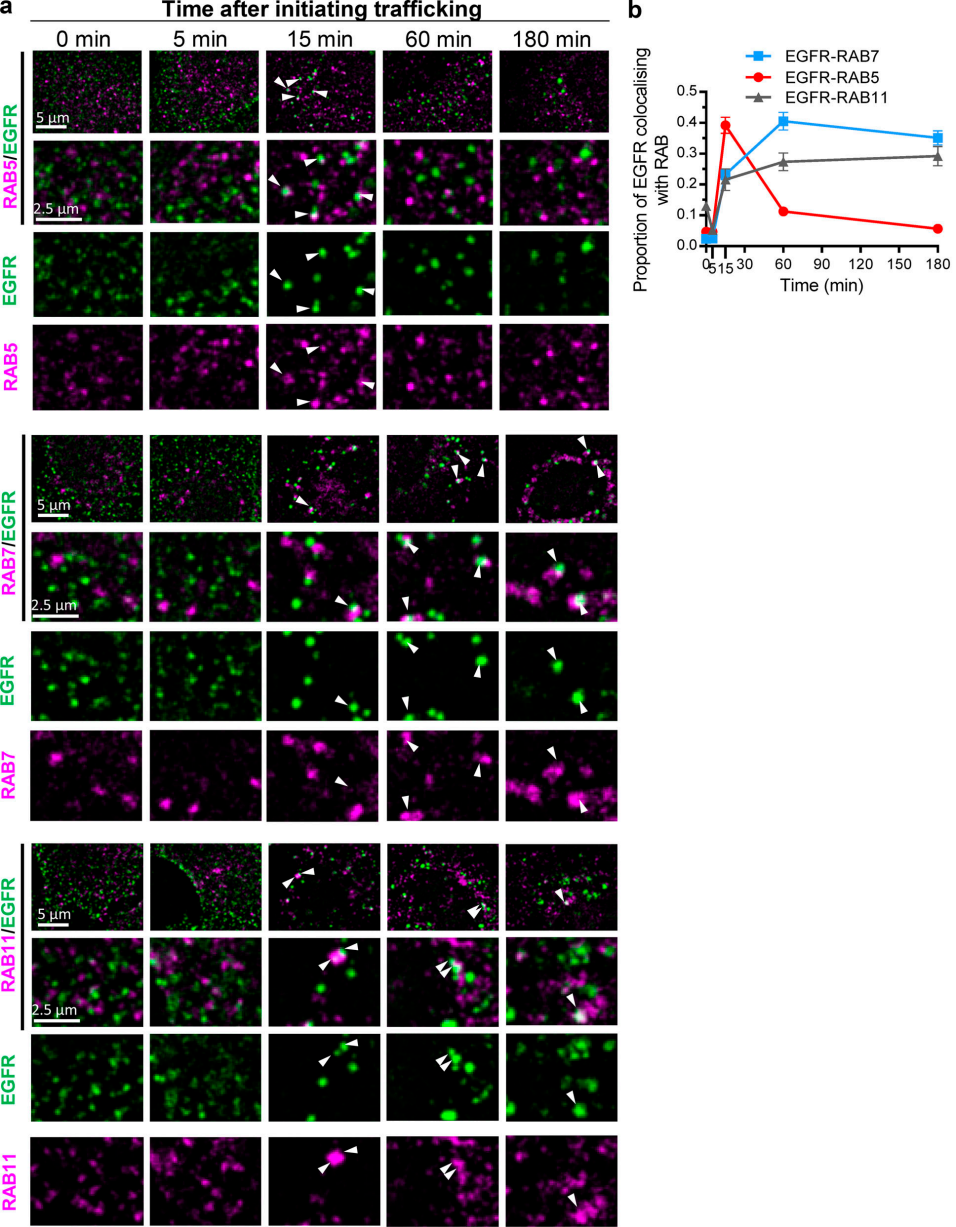
**Time courses of association of RAB 5, 7, or 11 with EGFR-containing endosomes. (a)** Representative low- and high-magnification and split-color confocal micrographs of EGFR-containing endosomes (EGFR, Alexa Fluor 594, false-colored green) costained with RAB5, RAB7, or RAB11 (Alexa Fluor 488, false-colored red), as indicated, at the indicated times after initiating EGFR trafficking. Arrowheads indicate colocalization. **(b)** Time course of colocalization of EGFR-containing endosomes with RAB5, RAB7, or RAB11 quantified from confocal micrographs; mean ± SEM; *n* > 30 cells per time point.

**Figure S5.**
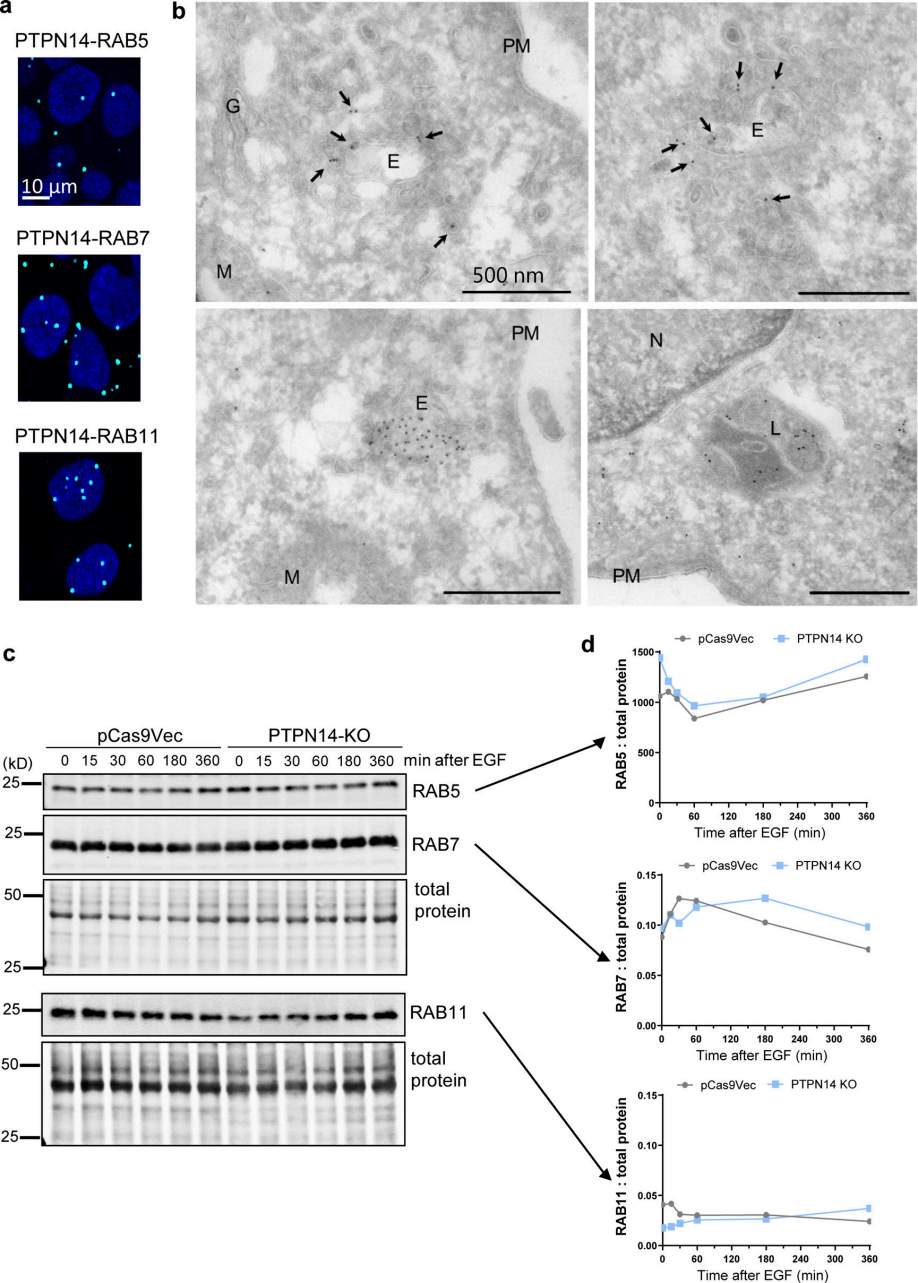
**PTPN14 colocalizes with endosomes, but pY374-PKCδ level does not affect total RAB protein expression. (a)** Colocalization of PTPN14 with the indicated RABs determined by PLA. Cells were stimulated with 20 ng/ml EGF for 15 min, fixed, and stained with anti-rabbit PTPN14 and anti-mouse RAB5, RAB7, or RAB11, followed by proximity ligation using anti-rabbit PLUS and anti-mouse MINUS probes. **(b)** Electron micrographs of immunogold-labeled PTPN14 in various intracellular organelles as indicated. Immuno-EM localization of FLAG-tagged PTPN14. MDA-MB-231 cells were incubated with dox to induce expression of FLAG-tagged PTPN14 and were then fixed and processed for immuno-EM. Labeling was evident on putative early endosomal compartments (E) and associated membranes (arrows) as well as late endosomal compartments (L). Mitochondria (M) showed variable labeling. N, nucleus; PM, plasma membrane; G, Golgi. **(c and d)** Representative Western blot (c) and quantitation relative to total protein (d) from *n* = 3 independent experiments showing RAB5, RAB7, and RAB11 protein levels in pCas9Vec and PTPN14-KO cells stimulated with EGF for the indicated times.

The recruitment of specific RABs is essential for defining the function of specific endosomal compartments (Zerial and McBride, 2001; Gruenberg, 2001; Huotari and Helenius, 2011). Early after endocytosis, RAB5 is recruited to early/sorting endosomes, and this is essential for the subsequent recruitment of RAB7, which is required for maturation of early to late endosomes. RAB11 is also recruited to early/sorting and recycling endosomes and is involved in recycling of RTKs back to the plasma membrane (Rink et al., 2005; Poteryaev et al., 2010; Vonderheit and Helenius, 2005; Goh and Sorkin, 2013). Comparing the kinetics of association of EGFR-containing endosomes with pY374-PKCδ or PTPN14 (Figs. 6 and 7) to that of EGFR-containing endosomes with RAB5, RAB7, and RAB 11 (Fig. 8), it was evident that the kinetics of pY374-PKCδ association with EGFR-containing endosomes was similar to that of RAB5 recruitment to the EGFR-containing endosomes, whereas PTPN14 association was more akin to that of RAB7/RAB 11. Of particular note is that 60 min after EGF stimulation, RAB5 association with endosomes was at a minimum, while RAB7 was at a maximum, indicating that a complete RAB5-to-RAB7 switch had occurred. This coincided with the minimum level of pY374-PKCδ and maximum level of PTPN14 associated with EGFR-containing endosomes, consistent with the notion that dephosphorylation of pY374-PKCδ by PTPN14 is necessary for the RAB5-to-RAB7 switch. The long period of association of PTPN14 with EGFR-containing endosomes also suggests that it remains associated with early endosomes through their maturation into late endosomes. In support of this, PTPN14 is colocalized with endosomes containing RAB5, RAB11, or RAB7 as determined by PLAs (Fig. S5 a) and is associated with various endosomal membrane compartments when visualized by immunogold EM (Fig. S5 b).

### pY374-PKCδ stabilizes the association of RABs 5 and 7 with EGFR-containing endosomes

**Figure 9.**
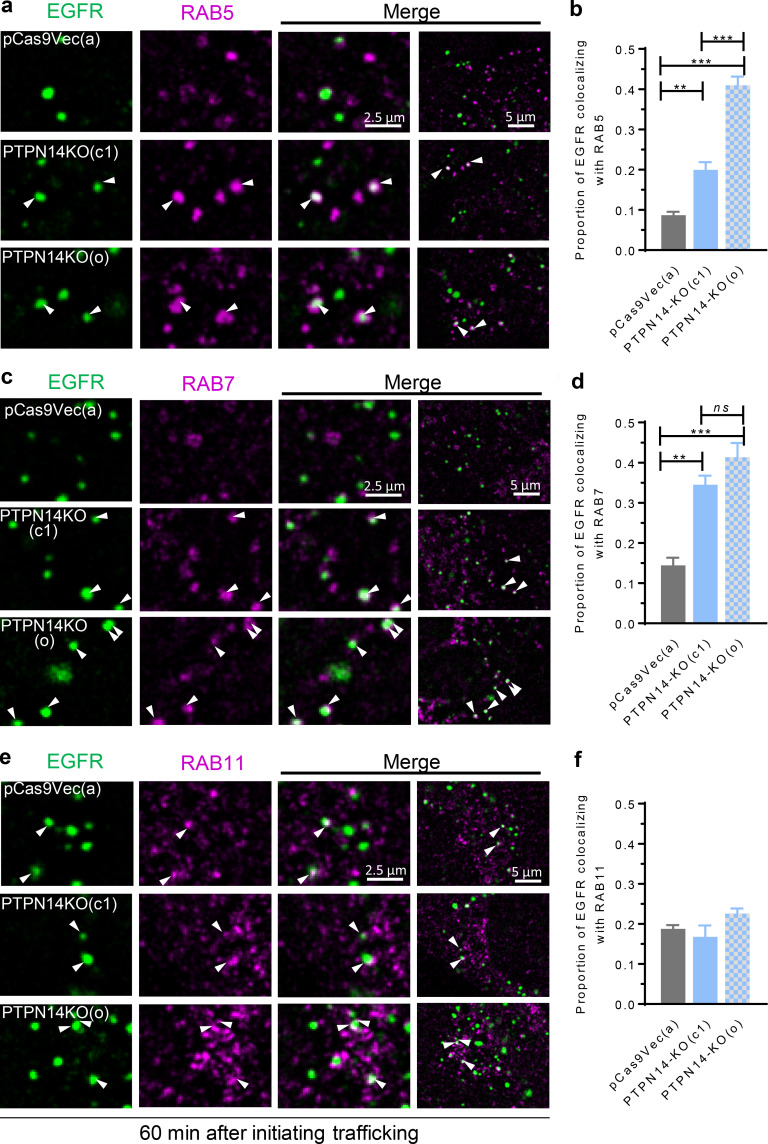
**pY374-PKCδ stabilizes association of RAB5 and RAB7 with EGFR-containing endosomes. (a–f)** EGFR-containing endosomes (Alexa Fluor 488) and the indicated RABs (Alexa Fluor 594) in control pCas9Vec (clone a) or PTPN14-KO (two clones, c1 and o) BT-549 cells were fluorescently tagged as described in Fig. 6. Representative confocal micrographs of EGFR-containing endosomes costained with RAB5 (a), RAB7 (c), or RAB11 (e) 60 min after inducing endocytosis with 20 ng/ml EGF (arrowheads indicate colocalization), and quantified data of colocalization of EGFR-containing endosomes with RAB5 (b), RAB7 (d), or RAB11 (f) from confocal micrographs (mean ± SEM; *n* > 30 cells; ns, not significant; **, P < 0.01; ***, P < 0.001 using one-way ANOVA with Bonferroni multiple comparisons post-test).

We next determined whether pY374-PKCδ level regulates the association of specific RABs with EGFR-containing endosomes in PTPN14-KO cells. While expression of RAB5, RAB7, and RAB11 was similar in control and PTPN14-KO cells (Fig. S5, c and d), colocalization of EGFR-containing endosomes with RAB5 or RAB7 was two- to fourfold higher in PTPN14-deficient cells 60 min after initiating trafficking (Fig. 9, a–d). Consistent with the lack of effect of pY374-PKCδ on RAB11 recruitment to Rab5-positive endosomes (Fig. 5, a and b), association of RAB11 with EGFR-containing endosomes was also unaffected by increased pY374-PKCδ (Fig. 9, e and f). These data are also consistent with the notion that increased pY374-PKCδ stabilizes the association of both RAB5 and RAB7 with EGFR-containing endosomes and support our earlier data (Fig. 5), which showed that high pY374-PKCδ is inhibitory to RAB5 release after RAB7 recruitment to endosomes.

### High pY374-PKCδ correlates with increased numbers of RAB5-RAB7 transitional endosomes and receptor activation in breast cancer

**Figure 10.**
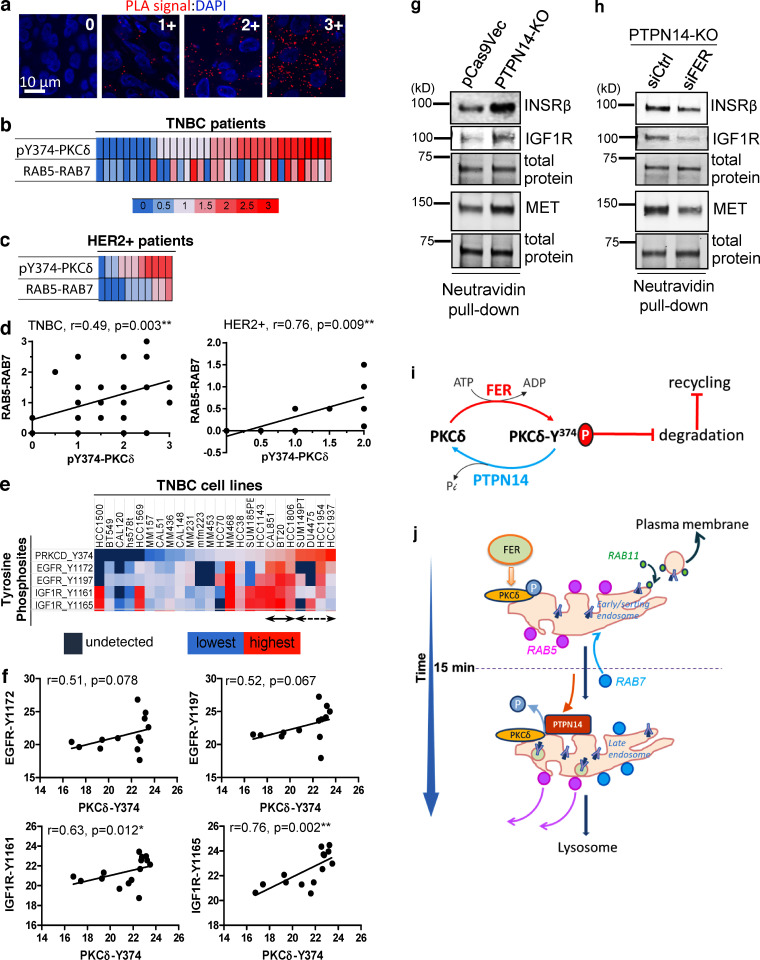
**High pY374-PKCδ is associated with dysregulated RTK trafficking and increased signaling in breast cancer. (a–d)** Increased pY374-PKCδ is correlated with increased RAB5-RAB7 colocalization in TNBC and HER2^+^ breast cancers. A TMA of formalin-fixed, paraffin-embedded samples from *n* = 35 TNBC patients and *n* = 11 HER2^+^ breast cancer patients (two cores per patient) were stained for pY374-PKCδ (using rabbit anti-pY374-PKCδ and mouse anti-PKCδ Abs) or RAB5-RAB7 colocalization (using rabbit anti-RAB5 and mouse anti-RAB7 Abs) by PLA (red dots) and counterstained with DAPI (blue). Data show representative confocal images of PLA signal strengths on a scale of 0–3+ (a) and heatmaps of pY374-PKCδ signals (upper panels) and RAB5-RAB7 colocalization signals (lower panels) in triple-negative (b) and HER2^+^ (c) breast cancers. Correlation between pY374-PKCδ levels and RAB5-RAB7 colocalization levels (d) in TNBC (left) and HER2^+^ breast cancer (right) were analyzed by Spearman linear regression analysis. **(e and f)** Elevated pY374-PKCδ correlates with RTK activation in TNBC cell lines. Phosphorylation levels of Y374-PKCδ, Y1172-EGFR, Y1197-EGFR, Y1161-IGF1R, and Y1165-IGF1R were quantified by phosphoproteomics from 24 TNBC lines and presented as a heatmap (e) or as a correlation between pY374-PKCδ expression and pY1172-EGFR, pY1197-EGFR, pY1161-IGF1R, and pY1165-IGF1R (f), using Spearman linear regression analysis. **(g and h)** pY374-PKCδ–induced cell surface RTK expression is dependent on FER. Western blots of cell surface biotinylated proteins from control (pCas9Vec) or PTPN14-KO cells (g) or from PTPN14-KO cells transfected with control (siCtrl) or FER siRNAs (siFER; h), pulled down using NeutrAvidin Sepharose and probed with the indicated antibodies. Data showing representative blots from *n* = 3 biological replicates. **(i)** Proposed model for the FER-PKCδ-PTPN14 axis in regulating RTK recycling and degradation. Phosphorylation and dephosphorylation of Y374-PKCδ by FER and PTPN14, respectively, regulates degradation and recycling. **(j)** Proposed model of recruitment of pY374-PKCδ, PTPN14, RAB5, RAB11, and RAB7 to the endosomes. Upon ligand binding, Y374-PKCδ is phosphorylated by FER and recruited to early endosomes containing RAB5. PTPN14 is recruited later, which coincides roughly with RAB7 recruitment, causing dephosphorylation of pY374-PKCδ, which facilitates RAB5 shedding from the transient RAB5-RAB7–positive transitional endosome to enable its maturation into late endosome and fusion with lysosome leading to degradation of RTK cargo.

High expression of EGFR is frequently associated with TNBC (Costa et al., 2017), and we previously identified a correlation between high PKCδ expression and overall decreased survival of luminal A breast cancer patients (Belle et al., 2015). This prompted us to investigate whether there is evidence of correlation between high levels of pY374-PKCδ and the occurrence of RAB5-RAB7–positive transitional endosomes, indicative of trafficking defects, in breast cancer. To do this, we used PLAs to analyze a tissue microarray (TMA) containing specimens from 35 TNBC and 11 HER2^+^ patients for pY374-PKCδ levels (using a pY374-PKCδ and PKCδ Ab pair) and degree of RAB5-RAB7 colocalization (using a RAB5 and RAB7 Ab pair). Using a 0–3 grading system (Fig. 10 a) to score the level of PLA signal, we found a significant correlation between pY374-PKCδ levels and the presence of RAB5-RAB7–positive transitional endosomes in TNBC (Fig. 10, b and d) and HER2^+^ patients (Fig. 10, c and d). Our data show that in each cancer subtype, a cohort of patients exist that exhibit high levels of both pY374-PKCδ and RAB5-RAB7 transitional endosomes, suggesting these patients exhibit altered endosomal RTK trafficking correlated with high levels of pY374-PKCδ.

To determine whether pY374-PKCδ expression was related to RTK activation in TNBC, we analyzed data from global mass spectrometry (MS)-based phosphotyrosine profiling across a panel of TNBC cell lines. Of the 24 cell lines profiled, 11 showed elevated levels of pY374-PKCδ together with increased site-selective phosphorylation of EGFR or insulin-like growth factor 1 receptor (IGF1R), indicative of RTK activation (Zhang et al., 2011; Wolf-Yadlin et al., 2006), with eight cell lines showing increased levels of tyrosine phosphorylation of both RTKs (Fig. 10, e and f). Despite the small dataset used, these correlations approached significance for EGFR sites and were highly significant for IGF1R sites. These data suggest that enhanced recycling of both EGFR and IGF1R due to increased pY374-PKCδ may increase the proportion of cell surface receptors available for ligand binding and receptor activation in these cells. We confirmed that elevated pY374-PKCδ levels (induced by PTPN14 deficiency) indeed led to an increase in cell surface expression of IGF1R, as seen for EGFR, and found that cell surface expression of insulin receptor β and the proto-oncogene MET was also elevated in these cells (Fig. 10 g). Importantly, the increase in cell surface presentation of all three receptors was rescued by knocking down FER (Fig. 10 h). These data provide evidence that the phosphorylation status of Y374-PKCδ, regulated by FER and PTPN14, can regulate the endosomal trafficking of multiple RTKs in breast cancer cells.

## Discussion

PKCδ is a member of the novel subfamily of the PKCs (Newton, 2018) with multiple functions ranging from immunodeficiency (Salzer et al., 2013) to subcellular localization–dependent pro- or antiapoptotic effects (Gomel et al., 2007). Further indications of the importance of the subcellular localization of PKCδ to its function was recently demonstrated by a study reporting its role in EGFR TK inhibitor resistance when localized in the nucleus in non–small cell lung cancer (Lee et al., 2018). In addition to being regulated by diacylglycerol, like other members of the PKC family, the catalytic activity of PKCδ is also regulated by phosphorylation on Ser and Thr residues and to a lesser extent on Tyr residues (Steinberg, 2004). Phosphorylation on Y374 of PKCδ has been reported (Olsen et al., 2010), and we have previously identified phospho-Y374-PKCδ as a substrate of PTPN14 (Belle et al., 2015), but the functional consequences of phosphorylation on Y374 was unknown, nor has the kinase that phosphorylates it been identified. We now show, for the first time, that Y374 on PKCδ is phosphorylated by the tyrosine kinase FER and that the resultant pY374-PKCδ compromises early to late endosome maturation and consequently impairs degradation of RTKs, thereby promoting recycling back to the cell surface to increase signaling and anchorage-independent cell growth, a surrogate measure for tumor-forming potential. We therefore propose that the regulation of Y374-PKCδ phosphorylation by a balance of FER and PTPN14 activities controls the balance between receptor degradation and recycling (Fig. 10 i), which ultimately determines the magnitude and duration of signaling downstream from these receptors. Although we have focused on the EGFR as a model RTK to study the effects of dysregulation of this axis on endosomal trafficking, our previous data on VEGFR3 (Belle et al., 2015) and the effects on IGF1R, insulin receptor β, and MET in this study indicate that a broad range of RTKs are likely to be regulated by this axis.

During their maturation to late endosomes, the RAB5-positive early endosomes acquire RAB7 and subsequently release RAB5, an obligatory step (Rink et al., 2005; Huotari and Helenius, 2011), to form a mature late endosome capable of delivering its contents to the lysosome for degradation. During this RAB5-to-RAB7 switching process, there is a transient interim period wherein this compartment displays both RAB5 and RAB7 on the endosomal membrane, indicative of a transitional status (Rink et al., 2005). Our data support a model (Fig. 10 j) whereby phosphorylation of Y374-PKCδ by FER occurs soon after ligand stimulation, and the pY374-PKCδ rapidly associates with the endocytosed EGFR, with the maximum association coinciding roughly with maximum levels of RAB5 associated with the endocytosed EGFR. RAB7 and PTPN14 recruitment then follow, with PTPN14 recruitment causing dephosphorylation of PKCδ, a step essential to promote RAB5 release to facilitate fusion to the lysosome, thus delivering the EGFR for degradation. Although our kinetic analysis does not allow us to assign the precise order of RAB7 and PTPN14 recruitment to the EGFR-containing endosomes, our observation that PTPN14 loss increases RAB7 recruitment to the EGFR-containing endosomes (Fig. 9, c and d) suggests that RAB7 recruitment may be inhibited by PTPN14, and therefore most likely occurs before or concurrently with recruitment of PTPN14. This being the case, our data would suggest that the recruitment of RAB7 without the subsequent recruitment of PTPN14 is inadequate to release RAB5 and complete the RAB5-RAB7 switch. Taken together, our data indicate that the newly identified FER-PKCδ-PTPN14 axis constitutes a critical regulatory mechanism that controls the rate of RAB5-RAB7 switching, such that when this axis is dysregulated to favor hyperphosphorylation of Y374-PKCδ, the RAB5-RAB7–positive transitional endosomes persist, leading to prolonged retention of RTKs. We contend that prolonging the retention of RTKs in the RAB5-RAB7–positive transitional endosomes presents greater opportunity for the continued recycling of endocytosed receptors, as the presence of RAB5 is not inhibitory to recycling (Sönnichsen et al., 2000).

One question that remains is the precise nature of these stabilized RAB5-RAB7–positive transitional endosomes and the mechanism by which the receptors normally destined for degradation may be preferentially redirected for recycling from this compartment. Since we see no evidence of altered receptor ubiquitination, it may be that sorting of the receptors into ILVs, which begins in the early endosomes, proceeds as normal and that the receptors are subsequently rescued from their degradative fate through back-fusion of the ILVs with the limiting membrane of the transitional endosomes (Bissig and Gruenberg, 2014). Alternatively, despite receptor ubiquitination occurring, the receptors may be preferentially sorted into recycling domains of this compartment, at the expense of active sequestration into ILVs (MacDonald et al., 2018), perhaps via ubiquitination or other posttranslational modification of Hrs or other components of the machinery responsible for recognition or sorting of the ubiquitinated cargo (Hoeller et al., 2006; Sun et al., 2013). Indeed, it has been noted that the SNX-BAR (sorting nexin proteins containing a Bin-Amphiphysin-Rvs domain)–dependent sorting of cargo away from the degradative pathway and into the recycling pathway occurs predominantly from this transient RAB5-RAB7–positive transitional endosome stage (van Weering et al., 2012). It is also possible that these transitional endosomes may represent a recycling-competent late endosome/lysosomal compartment, such as that shown to be involved in recycling of endocytosed α5β1 integrin and membrane type 1 matrix metalloproteinase in ovarian and TNBC cells (Dozynkiewicz et al., 2012; Macpherson et al., 2014). This compartment is known to be positive for RAB7 and LAMP1 (with or without a requirement for RAB25/RAB11c), but colocalization of RAB5 was not determined in these studies. Understanding the nature of this stabilized RAB5-RAB7–positive endosome and the mechanisms involved in recycling from this compartment, as well as the range of cargo mediated by this pathway, will be of great interest in future studies.

The pathophysiological significance of the FER-PKCδ-PTPN14–mediated pathway we have identified is highlighted by our finding that high levels of pY374-PKCδ correlate with high levels of the RAB5-RAB7 transitional phenotype in triple-negative and HER2^+^ breast cancers. We suggest that hyperphosphorylation of Y374-PKCδ and high levels of the RAB5-RAB7–positive transitional endosomes are potential biomarkers for identifying patients with dysregulation of the FER-PKCδ-PTPN14 axis that favors recycling of RTKs back to the plasma membrane to continue signaling. Cancers with dysregulation of this axis are likely to be more aggressive due to the higher availability of multiple RTKs on the plasma membrane for ligand binding and therefore not amenable to treatments targeting single RTKs. Such cancers may include those with loss-of-function PTPN14 mutations, reported in colorectal (Wang et al., 2004), breast (Sjöblom et al., 2006), head and neck (Stransky et al., 2011), and liver (Li et al., 2011) cancers. Similarly in neuroblastoma, PTPN14 mutations are associated with disease relapse (Schramm et al., 2015), while high levels of FER expression have been detected in breast (high-grade basal/TNBC; Ivanova et al., 2013), clear cell renal cell carcinoma (Wei et al., 2013), and non–small cell lung cancer (Kawakami et al., 2013), where high FER expression predicts poorer survival outcomes.

In addition to EGFR, IGF1R and MET have also been reported to be amplified in TNBC (Koboldt et al., 2012), and all four RTKs that we have found to be regulated by pY374-PKCδ in TNBCs are up-regulated in the reprogrammed kinome following treatment with MEK-I, AKT-I, or TK inhibitor (Duncan et al., 2012). Interestingly, FER activity is also up-regulated in the C3Tag mouse model of TNBC following treatment with MEK-I (Duncan et al., 2012). Based on our data, we suggest that it may also be possible to manipulate the balance of the FER-PKCδ-PTPN14 axis to simultaneously inhibit downstream signals emanating from multiple RTKs to resensitize TNBC or other cancers that have developed adaptive resistance to MEK-I, ALK-I, or BRAF-I through up-regulation of multiple RTKs, without the need to determine which, and how many, RTKs are hyperactivated.

## Materials and methods

### Antibodies

The antibodies used in this study were as follows: anti-EGFR (D38B1) rabbit mAb (4267), anti–HA-tag (6E2) mouse mAb (2367), anti-EGFR (D38B1) rabbit mAb (4267), anti-insulin receptor β (L55B10) mouse mAb (3020), anti–IGF-I receptor β (D23H3) rabbit mAb (9750), anti-MET (D1C2) rabbit mAb, anti-FER (5D2) mouse mAb (8198), anti-RAB5 (C8B1) rabbit mAb (3547), anti-RAB11 (D4F5) rabbit mAb (5589), anti-RAB7 (D95F2)XP(R) rabbit mAb (9367), anti–phospho-p44/42 MAPK (T202/Y204) rabbit mAb (9101), and anti-p44/42 MAPK (Erk1/2; L34F12) mouse mAb (4696); (all from Cell Signaling Technology); anti-Vinculin (SPM227) mouse mAb (ab18058; Abcam), anti–EGFR-ECD mouse mAb (Ab-3, clone EGFR.1; ms311; Thermo Fisher Scientific), anti-PKCδ mouse mAb (610398; BD Transduction Laboratories), anti-RAB7 (D-4) mouse mAb (sc-271608, IF; Santa Cruz Biotechnology), and anti-myc (9E10 clone) mouse mAb (generous gift of Dr. Timothy Hercus, Centre for Cancer Biology, an alliance of SA Pathology and University of South Australia, Adelaide, Australia).

### siRNAs

The following siRNAs were used in this study: PTPN14 siRNA (Ambion Silencer Select 4390826; 5′-CCA​CGA​AGU​UUC​GAA​CGG​ATT-3′); PKCδ, four individual siRNAs (Ambion Silencer Select AM16708; 5′-GGU​UCA​CAA​CUA​CAU​GAG​CTT-3′); GenePharma 2607 (5′-GCA​CAA​GCU​GUU​UGA​ACC​AUU-3′), 2510 (5′-CUG​UAU​AUA​UUG​CUC​AGU​AUU-3′), and 2376 (5′-CUG​UGA​ACU​GUG​UGU​GAA​UUU-3′); FER siRNAs, a pool of four individual siRNAs (Dharmacon Smartpool 003129-02; 5′-GGA​GUG​ACC​UGA​AGA​AUU​C-3′, 5′-UAA​AGC​AGA​UUC​CCA​UUA​A-3′, 5′-GGA​AAG​UAC​UGU​CCA​AAU​G-3′, and 5′-GAA​CAA​CGG​CUG​CUA​AAG​A-3′) and two individual siRNAs (siGenome D-003129 07 0005; 5′-GGA​AAG​UAC​UGU​CCA​AAU​G-3′) and D003129 08 0005 (5′-GAA​CAA​CGG​CUG​CUA​AAG​A-3′); and a negative control siRNA (Ambion Silencer Select negative control #2, 4390847).

### Cell lines and culture

The BT549, BT20, DU4475, HCC38, HCC70, HCC1500, HCC1569, HCC1954, HCC1806, HCC1143, HCC1937, HS578T, MDA-MB-157, MDA-MB-436, MDA-MB-453, MDA-MB-231, and MDA-MB-468 cell lines were purchased from the American Type Culture Collection; CAL51, CAL148, and CAL851 cells were obtained from Deutsche Sammlung von Mikroorganismen und Zellkulturen; SUM185PE and SUM149PT cells were purchased from Asterand Bioscience; MFM223 cells were purchased from Sigma-Aldrich; and CAL120 cells were a gift from Professor Elgene Lim from the Garvan Institute of Medical Research, Darlinghurst, NSW, Australia. All the above cell lines were cultured in RPMI supplemented with 10% FBS and 0.023 IU/ml (or 10 µg/ml) insulin. MDA-MB231(LM2) cells (a kind gift from Dr. Joan Massague, Sloan-Kettering Memorial Institute, New York, NY; Minn et al., 2005) were cultured in DMEM supplemented with 10% FBS.

### RT-PCR primers

RT-PCR primers were EGFR forward, 5′-CAG​TGG​CGG​GAC​ATA​GTC​AG-3′, and reverse, 5′-TTG​GTC​AGT​TTC​TGG​CAG​TTC​T-3′; and GAPDH forward, 5′-ACC​CAG​AAG​ACT​GTG​GAT​GG-3′, and reverse, 5′-CAG​TGA​GCT​TCC​CGT​TCA​G-3′.

### Generation of stable cell lines

#### PTPN14 KO cell lines

PTPN14-KO cells were generated using the CRISPR/Cas9 system (Ran et al., 2013) to disrupt PTPN14 expression. Briefly, guide sequences (top, 5′-CAC​CGA​CAC​GGC​GCT​ACA​ACG​TCC-3′, and bottom, 5′-AAA​CGG​ACG​TTG​TAG​CGC​CGT​GTC-3′, targeting exon 2 of ptpn14) were ligated into the pSpCas9(BB)-2A-Puro vector (PX459, 48139; Addgene) and transfected into BT-549 cells. 24 h after transfection, cells were selected with 2 µg/ml Puromycin for 48 h. Individual clones were generated and screened for loss of PTPN14 expression by Western blot analysis.

#### Generation of dox-inducible WT and Y374F-PKCδ BT-549 cell lines

A C-terminal myc-tagged PKCδ (PKCδ-myc) cDNA encoding the entire open reading frame was purchased from Sino Biologicals (HG 10769-CM). The Y374F mutation was generated by QuickChange (Stratagene) site-directed mutagenesis according to the manufacturer’s instructions. The WT and Y374F-PKCδ-myc cDNAs were subcloned into pInducer20 lentiviral vector (44012; Addgene; Meerbrey et al., 2011), and the resulting plasmids were sequenced to verify the sequences of the inserts. Lentiviruses were generated and used to transduce BT-549 or PTPN14-KO cells, and pools or clones of neomycin-resistant cells were collected after selection.

### Western blots and quantitation of proteins

After the indicated treatments, lysates in Laemmli buffer were resolved on SDS-PAGE using the TGX stain-free system (Bio-Rad), which has a proprietary stain embedded to stain total proteins loaded. Total protein in the gel before transfer and protein on the membrane after transfer were detected using the ChemiDocTouch or ChemiDoc MP system. Membranes were probed with the indicated antibodies and visualized using the Odyssey imaging system (Licor) or ChemiDocMP system (Bio-Rad). The sum of the intensities of multiple protein bands from grayscale images of total protein loaded (stained and scanned as described above), and specific proteins transferred to membranes detected by antibodies, were quantified using ImageJ (National Institutes of Health). In some instances, vinculin, as indicated, was used as a surrogate for normalizing total protein loaded.

### Fluorescence and bright-field microscopy

For immunofluorescence of permeabilized samples, cells were fixed with 3.7% PFA solution in PBS, permeabilized with 0.1% Triton X-100, and blocked with 2% BSA in TBS/0.1% Triton X-100 before applying indicated primary antibodies and corresponding fluorescently labeled secondary antibodies. For surface staining, in unpermeabilized samples, cells were fixed with 3.7% PFA solution on ice for 5 min followed by 10 min at RT and blocked with 2% BSA in TBS before applying the indicated primary antibodies and corresponding fluorescently labeled secondary antibodies. The fluorochromes used in this study were DAPI, Alexa Fluor 488, and Alexa Fluor 594 (Thermo Fisher Scientific), as indicated in the figures. All microscopy samples were mounted using ProLong Gold Antifade Mountant with DAPI (Thermo Fisher Scientific).

Fluorescence images were captured at ambient temperature using confocal laser scanning microscopes (either a Zeiss LSM 700 with C-Apochromat 40×/NA 1.20 water-immersion objective or Leica TSC SP8 with HC PL APO CS2 40×/NA 1.1 water-immersion objective). Images were acquired using LAS X software (saved in LIF format) on the Leica SP8 or Zen Black software on the Zeiss LSM700 (saved in CZI format). Image processing was performed using Fiji/ImageJ.

For quantification, a minimum of 10 z-slices were taken per field of view, with a minimum of four fields of view per condition. Images used for comparisons were acquired using identical acquisition settings and postacquisition processing. Images shown in the figures were brightness/contrast adjusted to allow the best visualization of signal, guided by histograms for the stack under the Analyze menu to ensure the fluorescence signals were within the full dynamic range without saturation of pixel intensities. Images shown were background subtracted by subtracting a duplicate stack processed by Gaussian blur (sigma radius 10) from the original image. After background subtraction, images were smoothed by applying Gaussian blur (sigma radius 0.75).

Colocalization analysis was quantified using the ImageJ JACoP plug-in to calculate Manders overlap coefficient (Bolte and Cordelières, 2006), where a coefficient of 1 equals 100% colocalization and 0 equals no colocalization, and the coefficient is determined as either tM1, which is the “summed intensities of pixels from the green image for which the intensity in the red channel is above zero,” to the “total intensity in the green channel”; tM2 is defined conversely for red. Stacks of a minimum of four fields of view and 10 slices per condition were background subtracted (using rolling ball algorithm). Within JACoP, identical thresholds were applied to control and treatment conditions at each time point. Phase-contrast images were taken using an Olympus CKX41 inverted microscope (using UPlanFL 4× NA 0.13 objective) equipped with an Olympus DP21 camera.

### Colony formation assays

In a 6-well tray, cells were seeded at 6,000 cells per well in 0.33% agarose in RPMI + 10% FBS overlaid on top of a base 0.5% agarose layer in RPMI. For EGF-treated wells, 20 ng/ml EGF was added to the cell-containing layer and replenished every 3 d. Colonies were grown at 37°C and 5% CO_2_ for the indicated time periods. Colonies were imaged and counted using ImageJ. Images were background subtracted using rolling ball algorithm, thresholded to highlight the colonies, and converted to a binary image (black colonies on white background). The analyze particles function was used to count number of colonies, either >1,000 or >500 px^2^.

### Cell surface staining of EGFR

Cells were seeded on fibronectin-coated chamber slides at 4 × 10^4^ cells/well for 24 h and starved in low-serum medium (RPMI + 0.1% FBS) for 20 h. Cells were then incubated with anti-EGFR-ECD antibody for 1 h at 4°C, washed with PBS on ice to remove unbound antibody, and incubated with Alexa Fluor 488–anti-mouse secondary antibody at 4°C for 1 h in low-serum medium. Cells were washed three times with PBS on ice, fixed using 3.7% PFA for 15 min, and washed twice with PBS before mounting using Prolong Gold with DAPI (Thermo Fisher Scientific). Total fluorescence was quantified using ImageJ. Images were background subtracted using rolling ball algorithm and thresholded to highlight the surface stain signal. Identical thresholds were used across treatment conditions being compared. Total fluorescence of the surface staining was quantified using integrated density measurement under the analyze particles function and divided by number of cells per field of view, as determined by DAPI staining. Surface staining was quantified for minimum of 85 cells per condition.

### Biotinylation of cell surface proteins

Cells were seeded at 7 × 10^5^ cells per well in 24-well trays for 24 h and starved in low-serum medium (RPMI + 0.1% FBS) for 20 h. On ice, cells were washed three times in cold PBS, pH 8.0, before biotinylation of cell surface proteins by the addition of 0.8 mM EZ-Link-Sulfo-NHS-SS-Biotin (Thermo Fisher Scientific) in cold PBS, pH 8. Cells were then incubated for 60 min at 4°C with agitation to allow biotinylation to occur, followed by three washes in PBS, pH 8. Cells were lysed on ice in NP-40 lysis solution (1% NP-40, protease inhibitor complete-mini-EDTA free [Roche], 10 mM NaVO_4_, 10 mM NaF, and 10 mM β-glycerophosphate). Biotinylated surface proteins were captured from cleared lysates by incubating with NeutrAvidin-agarose beads (Thermo Fisher Scientific) overnight on a rotating wheel at 4°C, and then washed three times in 1% NP-40 buffer. Proteins pulled down by NeutrAvidin agarose were resolved on SDS-PAGE using a TGX stain-free system (Bio-Rad). Total protein in the gel before transfer and on the membrane after transfer were detected using the ChemiDoc MP system (Bio-Rad). Membranes were probed with the indicated antibodies, visualized using the Odyssey imaging system (Licor) or ChemiDoc MP (Bio-Rad), and quantified using ImageJ.

### Endocytosis

Cells were starved in low-serum medium (RPMI + 0.1% FBS) for 20 h before incubation with mouse anti–EGFR-ECD to label cell surface EGFR in the presence of 20 ng/ml EGF for 1 h at 4°C. After washing in PBS on ice to remove unbound antibody and ligand, endocytosis was initiated by placing cells in warm (37°C) low-serum chase medium for ≤30 min. Cells were placed on ice to stop endocytosis at the indicated times after initiating endocytosis, acid washed (0.2 M acetic acid and 0.5 M NaCl, pH 2.5) for 5 min to remove any Ab bound to EGFR remaining on the cell surface, washed three times in PBS, and fixed using 3.7% PFA for 15 min. Permeabilized cells were incubated with anti-mouse–Alexa Fluor 488 for 1 h at RT to detect internalized EGFR, washed three times with TBS/0.1% Triton X-100, and then mounted using ProlongGold with DAPI. Total intracellular fluorescence was quantified using ImageJ.

### Recycling

Recycling was assayed using two independent protocols.

### Immunofluorescence detection of cell surface recycled EGFR with prebound EGFR-ECD antibody

wt-PKCδ-myc or YF-PKCδ-myc BT-549 cells were grown in the presence of dox (1.0 µg/ml) for 2–3 d. Cells were then starved in low-serum medium (RPMI + 0.1% FBS) for 20 h in the presence of dox. For the assay, cells were prelabeled with mouse anti-EGFR-ECD antibody in the presence of 20 ng/ml EGF for 60 min at 4°C, after which medium was replaced with warm low-serum medium, and the cells were transferred to 37°C for endocytosis to occur for 15 min. After the pulse of endocytosis, cells were placed on ice and acid washed in 0.2 M acetic acid and 0.5 M NaCl, pH 2.5, for 5 min to remove Ab bound to EGFR remaining on the cell surface, followed by four washes in low-serum medium. Cells were then incubated at 37°C for 30 min in low-serum medium without EGF to allow recycling of EGFR back to the cell surface. At the end of the recycling period, the cells were placed on ice and fixed with 3.7% formaldehyde. Cells were washed twice in TBS, blocked in TBS/2% BSA, incubated with anti-mouse–Alexa Fluor 594–conjugated secondary Ab to detect EGFR-ECD Ab-bound EGFR that had returned to the cell surface, and then mounted using ProlongGold with DAPI. To determine the pool of EGFR internalized after the 15-min endocytosis pulse, an additional well was subjected to the pulse of endocytosis and then fixed, permeabilized, blocked, and stained with anti-mouse–Alexa Fluor 594–conjugated secondary Ab. For each time point, a minimum of six fields of view were quantified. Mean fluorescence intensity was quantitated using the analyze particles function in ImageJ.

### Detection of recycled EGFR by cell surface biotinylation

wt-PKCδ-myc or YF-PKCδ-myc BT-549 cells were grown in the presence of dox (1.0 µg/ml) for 2–3 d. Cells were then starved in low-serum medium (RPMI + 0.1% FBS) for 20 h in the presence of dox. For the assay, cells (except preendocytosis samples) were preloaded with 20 ng/ml EGF for 60 min at 4°C, after which medium was replaced with warm low-serum medium, and the cells were transferred to 37°C for endocytosis to occur for 30 min. Cells were washed three times with low-serum medium on ice, and then chased with warm low-serum medium at 37°C for the indicated times to allow receptor recycling. After the indicated times, cells on ice were washed three times in cold PBS, pH 8.0, before biotinylation of cell surface proteins by the addition of 0.8 mM EZ-Link-Sulfo-NHS-SS-Biotin (Thermo Fisher Scientific) in cold PBS, pH 8. Cells were incubated for 60 min at 4°C with agitation to allow biotinylation to occur, followed by three washes in PBS, pH 8. Cells were lysed on ice in NP-40 lysis solution (1% NP-40, protease inhibitor complete-mini-EDTA free [Roche], 10 mM NaVO_4_, 10 mM NaF, and 10 mM β-glycerophosphate). Biotinylated surface proteins were captured from cleared lysates by incubating with NeutrAvidin-agarose beads (Thermo Fisher Scientific) overnight on a rotating wheel at 4°C, and then washed three times in 1% NP-40 buffer. Proteins pulled down by NeutrAvidin agarose were resolved on SDS-PAGE using a TGX stain-free system (Bio-Rad). Total protein in the gel before transfer and on the membrane after transfer were detected using the ChemiDoc MP system (Bio-Rad). Membranes were probed with the indicated antibodies, visualized using the Odyssey imaging system (Licor) or ChemiDoc MP (Bio-Rad), and quantified using ImageJ.

### EGFR degradation

Cells were starved in low-serum medium (RPMI + 0.1% FBS) for 20 h and incubated with 100 µg/ml cycloheximide to inhibit protein synthesis for 30 min before addition of 20 or 100 ng/ml EGF to stimulate endocytosis. Cells were incubated at 37°C for the indicated times, up to 24 h. At the indicated times, cells were lysed with Laemmli buffer and lysates were resolved on SDS-PAGE and subjected to Western blotting. Membranes were probed with antibodies against EGFR and vinculin (as a loading control), visualized using Odyssey imaging system (Licor), and quantified using ImageJ.

### EGFR ubiquitination

1.2–1.8 × 10^6^ cells were transfected with the indicated siRNAs using RNAiMAX (Thermo Fisher Scientific) for 20 h. The next day, cells were transfected with HA-ubiquitin using Lipofectamine 2000 for 16 h. 48 h later, cells were starved in low-serum medium for 4 h and stimulated with 20 or 100 ng/ml EGF for the indicated times. After stimulation, cells were lysed in 1% NP-40 lysis solution (1% NP-40, protease inhibitor complete-mini-EDTA free [Roche], 10 mM NaVO_4_, 10 mM NaF, and 10 mM β-glycerophosphate). EGFR was immunoprecipitated from lysates using EGFR antibody (Ab-3 or D38B1) and protein-A-Sepharose. Immunoprecipitates were resolved on SDS-PAGE and subjected to Western blotting. Membranes were probed with antibodies against HA or ubiquitin and EGFR, visualized using the Odyssey imaging system (Licor) or ChemiDoc MP (Bio-Rad), and quantified relative to total protein using ImageJ.

### Colocalization studies

#### pY374-PKCδ, PTPN14, or RABs with endocytosed EGFR

Cells were starved in low-serum medium (RPMI + 0.1% FBS) for 20 h and then incubated with anti–EGFR-ECD antibody for 1 h at 4°C in the presence of 20 ng/ml EGF. After a PBS wash on ice to remove unbound antibody and ligand, cells were chased with warm low-serum medium to allow endocytosis to occur for the indicated times, after which cells were fixed using 3.7% PFA for 15 min. Permeabilized cells were incubated with RAB5, RAB7, or RAB11 antibodies overnight at 4°C followed by anti-mouse–Alexa Fluor 488 and anti-rabbit–Alexa Fluor 594 secondary Ab for 1 h at RT, washed three times in TBS/0.1% Triton X-100, and mounted using ProlongGold with DAPI.

#### RAB5 with RAB7 or RAB11

Cells were starved in low-serum medium (RPMI + 0.1% FBS) for 20 h and then incubated with 20 ng/ml EGF at 4°C. After a PBS wash on ice to remove unbound ligand, cells were chased with warm low-serum medium for the indicated times to allow endocytosis to proceed. Cells were then fixed using 3.7% PFA for 15 min. Permeabilized cells were incubated with mouse RAB5 (BD Bioscience) and rabbit RAB7 or RAB11 antibodies (Cell Signaling Technology) overnight at 4°C, washed, incubated with anti-mouse–Alexa Fluor 488 and anti-rabbit–Alexa Fluor 594 for 1 h at RT, washed three times in TBS/0.1% Triton X-100, and mounted using ProlongGold with DAPI. Fluorescence imaging, postimaging processing, and quantitation of colocalized pixels are as described in the fluorescence and bright-field microscopy section.

### EM

MDA-MB-231 cells expressing FLAG-tagged PTPN14 in a dox-inducible manner were treated with 10 ng/ml dox for 24 h and prepared for EM by sequential fixation in 2% PFA (5 min) and 4% EM-grade PFA (24 h) in 0.1 M phosphate buffer, pH 7.4. Cells were processed for frozen sectioning as described previously (Jung et al., 2018). Ultrathin thawed cryosections were labeled with antibodies to the FLAG tag, followed by 10 nm protein A-gold.

### PLA

PLAs were performed according to the manufacturer’s instructions (DuoLink; Sigma-Aldrich) on cultured BT-549 cells (fixed in 3.7% PFA for 15 min and permeabilized in TBS/0.1% Triton X-100) or TMA slides containing formalin-fixed, paraffin-embedded tumor sections from 35 TNBC and 11 HER2^+^ breast cancer patients (two cores per patient; Biomax) after processing and antigen retrieval (below). Slides were probed overnight with antibody pairs (either mouse anti-RAB5 [BD Bioscience] and rabbit anti-RAB7 [Cell Signaling Technology] or mouse anti-PKCδ [BD Bioscience] and rabbit anti–pY374-PKCδ). Slides were washed three times for 5 min with TBS containing 0.1% Triton X-100, and then incubated for 60 min at 37°C with anti-mouse MINUS and anti-rabbit PLUS PLA probes (Duolink; Sigma-Aldrich) diluted in block buffer. Slides were washed twice for 5 min in wash buffer A (0.01 M Tris, 0.15 M NaCl, and 0.05% Tween), incubated with ligation mix for 30 min at 37°C, washed twice for 2 min in wash buffer A, incubated with amplification mix for 100 min at 37°C, washed twice for 10 min in wash buffer B (0.2 M Tris and 0.1 M NaCl), washed 1 min in 0.01× wash buffer B, and mounted in Duolink mounting medium after counterstaining with DAPI. Slides were imaged on Olympus CV1000 or Leica SP8 confocal microscopes. PLA signals were quantified using the ImageJ find maxima tool under the Process menu. The noise tolerance was set to select only the PLA signals and applied to all treatment conditions. For tumor samples, care was taken to quantify only staining in tumor cells, avoiding the stroma: tumor and stromal regions were identified by comparisons with adjacent sections stained with hematoxylin and eosin and by DAPI staining.

### Processing and antigen retrieval of TMAs

Slides were sequentially incubated in xylene (twice for 5 min), ethanol (twice for 5 min), water (5 min), and PBS (twice for 5 min). Antigen retrieval was performed by boiling slides in 10 mM citric acid buffer for 15 min. Before staining, cooled slides were washed in water (three times for 5 min) and TBS (5 min), and then blocked using block buffer (10% goat serum in TBS/0.1% Triton X-100) for 1 h.

### Phosphoproteomics

Phosphoproteomics was undertaken essentially as described (Chew et al., 2020).

### Isolation of tyrosine-phosphorylated peptides for MS

TNBC cell lines were cultured until 80% confluent, washed twice with ice-cold PBS, and lysed directly in the dish with lysis buffer (6 M guanidine hydrochloride, 50 mM Tris-HCl, 1 mM sodium orthovanadate, 2.5 mM sodium pyrophosphate, and 1 mM β-glycerophosphate). Lysate protein (∼20 mg) was reduced with 5 mM TCEP at 37°C for 1 h and alkylated with iodoacetamide in the dark for 1 h. Lysates were then diluted 1:4 with ammonium bicarbonate (25 mM), followed by digestion with 1:200 LysC (Worthington) at RT for 4 h. Lysates were further diluted 10× from the original volume, followed by digestion with a 1:100 trypsin (Promega) at 37°C for 18 h. Tryptic digests were acidified with 10% trifluoroacetic acid (TFA) to pH 3, desalted on a C18 column (Thermo Fisher Scientific), and eluted with 0.1% TFA/40% acetonitrile (ACN). Peptides were dried in a SpeedVac and reconstituted in 1.8 ml of IAP wash buffer (1% *n*-octyl-β-d-glucopyranoside, 50 mM Tris-HCl, and 150 mM NaCl, pH 7.4). To capture tyrosine phosphorylated peptides, a mixture of phospho-tyrosine antibodies (P-Tyr-1000, 8954, Cell Signaling Technology; P-Tyr-100, 9411, Cell Signaling Technology; and P-Tyr-20, 610000, BD Biosciences) at 50 µg each, coupled to Sepharose beads (60-µl slurry; Rec-Protein G; Zymed), were incubated overnight with peptide samples at 4°C with gentle shaking. Antibody-coupled beads bound to peptides were washed three times with IAP buffer followed by three washes with water before elution of peptides with 110 µl of 0.15% TFA. Samples were then desalted on a C18 column (as described above) and dried in a SpeedVac. The dried peptides were reconstituted in 2% ACN/0.5% formic acid.

### MS analysis

Tyrosine-phosphorylated peptides captured from cell lysates were analyzed on an UltiMate 3000 RSLC nano LC system (Thermo Fisher Scientific) coupled to an LTQ-Orbitrap mass spectrometer (LTQ-Orbitrap; Thermo Fisher Scientific). Peptides were loaded via an Acclaim PepMap 100 trap column (100 µm × 2 cm, nanoViper, C18, 5 µm, 100 Å; Thermo Fisher Scientific) followed by peptide separation on an Acclaim PepMap RSLC analytical column (75 µm × 50 cm, nanoViper, C18, 2 µm, 100 Å; Thermo Fisher Scientific). For each liquid chromatography–tandem MS analysis, 1 µg of peptides measured by a Nanodrop 1000 spectrophotometer (Thermo Fisher Scientific) were loaded on the precolumn with microliter pickup. Peptides were eluted using a 2-h linear gradient of 80% ACN/0.1% formic acid at a flow rate of 250 nl/min using a mobile phase gradient of 2.5–42.5% ACN. The eluting peptides were interrogated with an Orbitrap mass spectrometer. The hyper-reaction monitoring data-independent acquisition (DIA) method was run using the following settings: a first survey scan (MS1) ranging from 400 to 1,220 m/z with a resolution of 35,000, using an automatic gain control target of 5e6 and a maximum ion injection time of 120 ms. MS1 was followed by tandem MS/MS (MS2) scans with a resolution of 35,000, using an automatic gain control target of 3e6 with automatic injection time. The MS2 scans were acquired through 19 overlapping DIA windows (30 to 222 Daltons), stepped collision energy of 22.5, 25, and 27.5%, and a 30-m/z isolation window.

### Hyper-reaction monitoring DIA data analysis

The spectral libraries were generated in Spectronaut 8 (Biognosys) using Maxquant database search output that was searched at 1% false discovery rate with the following settings: enzyme specificity was set to Trypsin/P, minimal peptide length of 6, and ≤3 missed cleavages allowed. The search criteria included carbamidomethylation of cysteine as a fixed modification; oxidation of methionine; acetyl (protein N terminus); and phosphorylation of serine, threonine, and tyrosine as variable modifications. The mass tolerance for the precursor was 4.5 ppm, and for the fragment ions, 20 ppm. The data-dependent acquisition files were searched against the human UniProt fasta database (v2015-08, 20,210 entries). The DIA data were then analyzed against the spectral library in Spectronaut 8, using the software’s default parameters. In brief, retention correction was set to dynamic indexed retention time (correction factor for window 1), decoy generation to scrambled (no decoy limit), enabling interference correction on MS2 level and 1% false discovery rate at peptide level. For quantification, only peptides with at least three transitions were selected and based on the top three proteotypic peptides for each protein. The data were normalized using the default setting.

### In vitro phosphorylation assay

1–10 ng of recombinant GST- or HIS-tagged FER catalytic domain (PV3806, Life Technologies; 14-605, Merck) and 0–20 ng recombinant GST-tagged PKCδ (ab60844; Abcam) were incubated for 30–60 min in 1× kinase assay buffer (25 mM Tris-Cl, pH 7.4, 10 mM MgCl_2_, 0.5 mM EGTA, 0.01% Triton X-100, and 2.5 mM DTT) in the presence or absence of 1 mM ATP (10–25 µl reaction volume). For plate assays, reactions were stopped by addition of 125 mM EDTA and transferred to a glutathione capture plate (315240; Pierce; 1 h at RT), followed by incubation with 1/400 anti-pY374 PKCδ antibody (Belle et al., 2015) and 1/2,000 anti-rabbit-HRP antibody and detection with TMB substrate solution (34021; Thermo Fisher Scientific [Pierce]). For Western blot, reactions were stopped by addition of 2× SDS load buffer (100 mM Tris-Cl, pH 6.8, 4% SDS, 20% glycerol, 0.005% bromophenol blue, and 10% 2-mercaptoethanol) and resolved on 10% polyacrylamide gels, followed by detection with the pY374 PKCδ antibody (Belle et al., 2015).

### Online supplemental material

Fig. S1 shows that loss of PTPN14 enhances cell surface expression of EGFR but not EGFR transcript or total protein. Fig. S2 shows that phosphorylation of Y374-PKCδ is required for EGFR recycling. Fig. S3 shows that loss of PTPN14 reduces degradation of EGFR but does not affect EGFR ubiquitination. Fig. S4 displays in silico identification of TKs that oppose the action of PTPN14 in regulating endosomal phenotype. Fig. S5 shows that PTPN14 colocalizes with endosomes but pY374-PKCδ level does not affect total RAB protein expression.
